# Bacopa Protects against Neurotoxicity Induced by MPP^+^ and Methamphetamine

**DOI:** 10.3390/molecules27165204

**Published:** 2022-08-15

**Authors:** Michela Ferrucci, Carla Letizia Busceti, Gloria Lazzeri, Francesca Biagioni, Stefano Puglisi-Allegra, Alessandro Frati, Paola Lenzi, Francesco Fornai

**Affiliations:** 1 Department of Translational Research and New Technologies in Medicine and Surgery, University of Pisa, Via Roma 55, 56126 Pisa, Italy; 2 I.R.C.C.S. Neuromed, Via Atinense 18, 86077 Pozzilli, Italy; 3 Neurosurgery Division, Department of Human Neurosciences, Sapienza University, 00135 Rome, Italy

**Keywords:** oxidative stress, ultrastructural morphometry, mitochondrial alterations, cell death, cell degeneration

## Abstract

The neurotoxins methamphetamine (METH) and 1-methyl-4-phenylpyridinium (MPP^+^) damage catecholamine neurons. Although sharing the same mechanism to enter within these neurons, METH neurotoxicity mostly depends on oxidative species, while MPP^+^ toxicity depends on the inhibition of mitochondrial activity. This explains why only a few compounds protect against both neurotoxins. Identifying a final common pathway that is shared by these neurotoxins is key to prompting novel remedies for spontaneous neurodegeneration. In the present study we assessed whether natural extracts from *Bacopa monnieri* (BM) may provide a dual protection against METH- and MPP^+^-induced cell damage as measured by light and electron microscopy. The protection induced by BM against catecholamine cell death and degeneration was dose-dependently related to the suppression of reactive oxygen species (ROS) formation and mitochondrial alterations. These were measured by light and electron microscopy with MitoTracker Red and Green as well as by the ultrastructural morphometry of specific mitochondrial structures. In fact, BM suppresses the damage of mitochondrial crests and matrix dilution and increases the amount of healthy and total mitochondria. The present data provide evidence for a natural compound, which protects catecholamine cells independently by the type of experimental toxicity. This may be useful to counteract spontaneous degenerations of catecholamine cells.

## 1. Introduction

In the present manuscript neuroprotective effects of extracts from *Bacopa monnieri* (BM) are investigated against two catecholamine neurotoxins, namely methamphetamine (METH) and 1-methyl-4-phenylpyridinium (MPP^+^). The former is a drug of abuse, while the latter is the active metabolite of the parkinsonism-inducing neurotoxin 1-methyl, 4-phenyl-1,2,3,6-tetrahydropyridine (MPTP). The neurotoxic effects of METH were originally reported for dopamine (DA) neurons in primates [1,2,3] and later on in rodents [4]. The toxicity of MPTP, which requires its conversion to MPP^+^, for DA neurons was similarly established in primates [5,6,7,8], and it was further demonstrated for DA and norepinephrine (NE) neurons in rodents [9]. The selective toxicity of both METH and MPP^+^ depends on their selective uptake within catecholamine neurons by specific transporters, namely the DA transporter (DAT) and the NE transporter (NET) [9,10,11,12,13,14,15,16,17]. This explains why DAT and NET inhibitors protect against neurotoxic effects induced by METH as well as MPP^+^ [8,9,13,15,18,19,20,21]. Although sharing similar targets (catecholamine neurons) and gateway mechanisms (catecholamine transporters), METH and MPP^+^ markedly differ in their molecular and cellular mechanisms to induce neurotoxicity. In fact, MPP^+^ mainly targets mitochondria to exert its neurotoxic effects [22], while METH neurotoxicity depends on oxidative species produced by massive DA release [23,24]. Thus, MPP^+^ disrupts the mitochondrial complex I [22,25,26,27], while METH disrupts the vesicular DA storage [28,29], leading to a massive increase in cytosolic DA and reactive oxygen species (ROS) levels [23,24,30,31]. Therefore, protection against MPP^+^ toxicity can be achieved by preventing its binding and molecular effects on mitochondria [32,33,34,35,36], while METH toxicity can be prevented by depleting DA stores [3,37,38,39,40].

The difference in the molecular mechanisms between MPP^+^ and METH explains why most compounds fail to produce a dual protection, while sometimes exerting opposite effects. For instance, while pre-treatment with DA-depleting agents such as reserpine and tetrabenazine prevents METH toxicity [37,40], it worsens MPTP toxicity [41]. Again, while deletion of the protein α-synuclein mitigates MPTP toxicity [42], it worsens METH toxicity [43]. These data indicate that, despite sharing the gateway to access catecholamine neurons, these neurotoxins partly engage different mechanisms to produce cell toxicity. Since both neurotoxins damage catecholamine cells [8,44], some final common pathways placed downstream of mitochondrial impairment or massive DA efflux are likely to take a leading role. These common downstream events shared by METH and MPP^+^ are expected to be relevant for the survival of catecholamine neurons and the onset of parkinsonian disorders since they do not depend on the specific mechanism of action of a specific neurotoxin.

In fact, when examining the downstream effects produced by either massive DA overflow (following METH) or severe mitochondrial impairment (following MPP^+^), the massive increase in ROS is a common consequence. In fact, METH produces a strong oxidation [45], which is mediated through DA oxidative metabolites [46,47,48,49]. Similarly, oxidative stress occurs along with ATP depletion as a consequence of mitochondrial inhibition by MPP^+^ [50,51,52].

In line with this, natural compounds, which occlude oxidative stress and counteract ROS formation, are expected to offer a dual protective action with potential relevance for the neurobiology of disease. The aims of the present study are to investigate whether: (i) BM is able to protect catecholamine cells against the toxicity produced by different neurotoxins, such as METH and MPP^+^; (ii) BM is able to suppress ROS production induced by METH and MPP^+^; (iii) BM is able to prevent mitochondrial alterations induced by METH and MPP^+^.

Recently, a number of natural compounds were shown to protect against METH or MPTP toxicity. Among them, extracts from the plant BM’s own significant antioxidant properties. In fact, a few studies indicate that extracts from BM may protect against MPP^+^ toxicity [53,54,55,56,57]. However, no study has so far used BM to concomitantly investigate the occurrence of toxicity with alterations in mitochondrial integrity by using ultrastructural morphometry. Similarly, no report is available where the measurement of ROS was combined with protection induced by BM. The present study extends the investigation of BM on MPP^+^ toxicity. Since MPP^+^ toxicity remains a model of DA damage with inherent bias to the specific mechanism of action of MPP^+^, the protective potential of BM was also assessed against METH toxicity. Therefore, in the present study, a wide range of doses of BM were administered to PC12 and SH-SY5Y cell cultures either in baseline conditions or before the administration of moderate neurotoxic doses of either MPP^+^ or METH. In these cell cultures, potential BM-induced neuroprotective or deleterious effects of BM were validated by multiple methods of light and electron microscopy along with the measurement of ROS production. Mitochondrial integrity, including the integrity of the mitochondrial membrane, was assessed by light and electron microscopy.

## 2. Results

### 2.1. Dose-Dependent Effects of Bacopa Monnieri (BM) on Cell Viability

A pilot series of experiments were aimed at analyzing a wide range of doses of BM alone in order to assess potential BM-induced neurotoxicity. This allowed a range of BM doses to be established below the toxic ones, to assess neuroprotection against either METH- or MPP^+^-induced toxicity. An assessment of cell viability was carried out in both PC12 and SH-SY5Y cell cultures by using different methods including Fluoro Jade B (FJB), which was recently validated to assess neurotoxicity in cell cultures [58]. Results concerning SH-SY5Y are shown in the Supplementary Figures.

**Figure 1:**
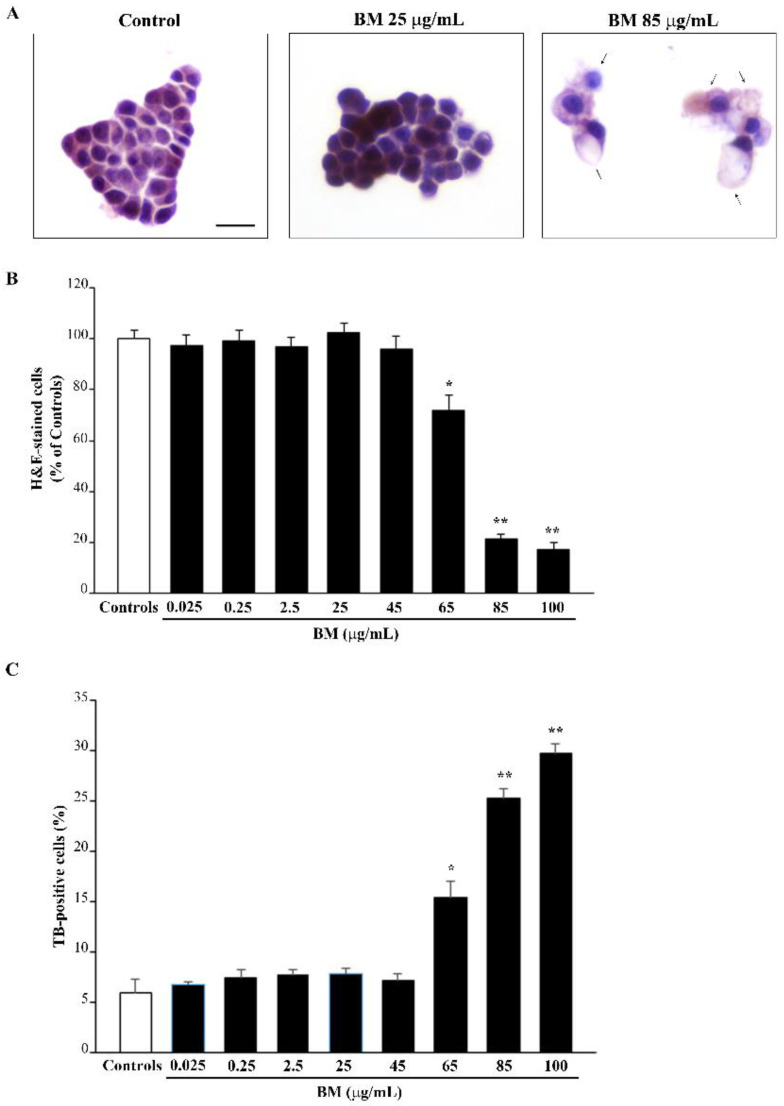
Dose–response of BM on H&E- and TB-stained cells. (**A**) Representative H&E staining, following various doses of BM. Arrows indicate cell alterations induced by high doses of BM. Graphs report the count of (**B**) H&E- and (**C**) TB-stained cells following increasing doses of BM (from 0.025 μg/mL up to 100 μg/mL). Values are given as mean percentage ± S.E.M. of cells from three independent experiments. * *p* < 0.05 compared with controls and BM up to 45 μg/mL; ** *p* < 0.05 compared with controls and BM up to 65 μg/mL. Scale bar = 12 μm.

**Figure 2:**
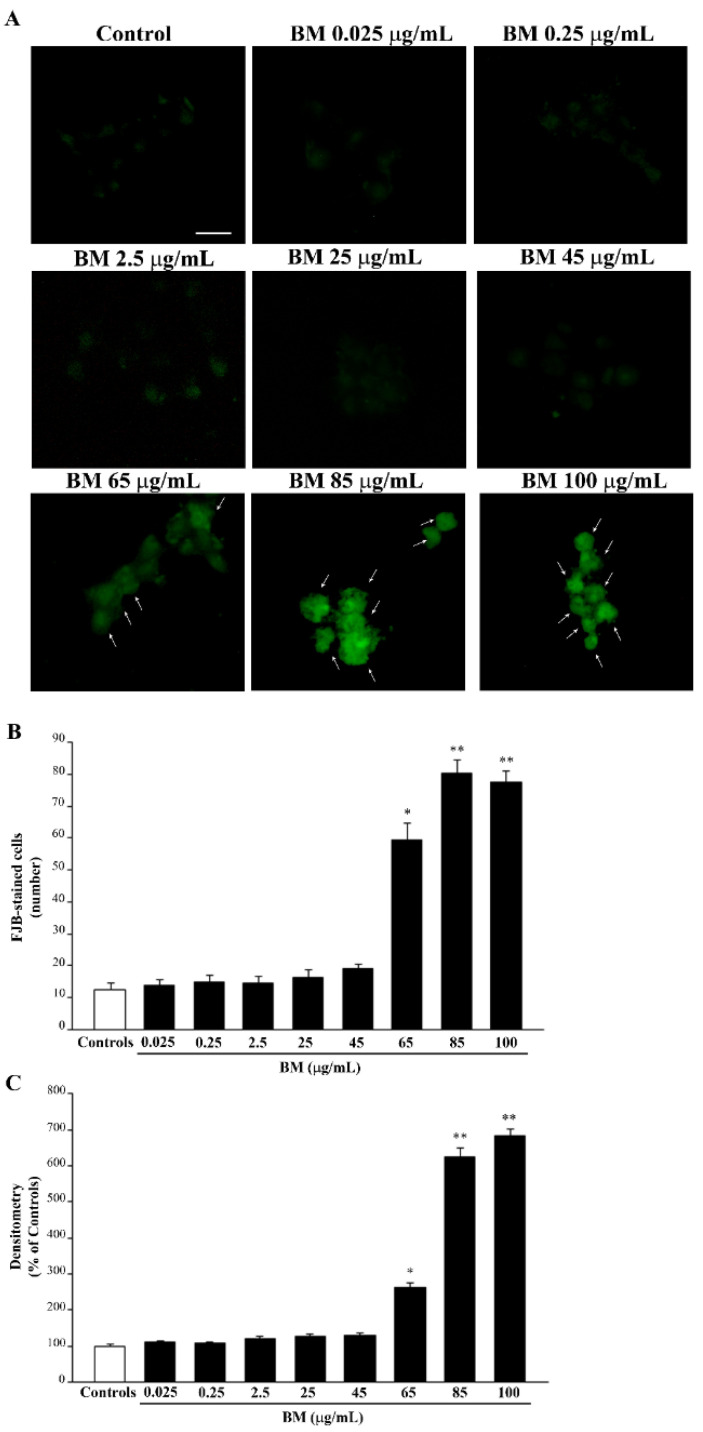
Dose–response of BM on FJB staining. (**A**) Representative pictures of FJB-stained PC12 cells after treatment with increasing doses (from 0.025 μg/mL up to 100 μg/mL) of BM for 72 h. Arrows indicate cells intensely stained with FJB. The graphs report the number (**B**) and the optical density (**C**) of FJB fluorescent cells treated with increasing doses of BM (from 0.025 μg/mL up to 100 μg/mL). Values are given as (**B**) mean ± S.E.M. of FJB-positive cells and (**C**) mean percentage ± S.E.M. of optical density (assuming controls as 100% density) from three independent experiments. * *p* < 0.05 compared with controls and BM up to 45 μg/mL; ** *p*< 0.05 compared with controls and BM up to 65 μg/mL. Scale bar = 12 μm.

A linear dose–response study was carried out with the following doses of BM, 0.025 μg/mL, 0.25 μg/mL, 2.5 μg/mL, 25 μg/mL, and 250 μg/mL. Since BM at the dose of 250 μg/mL produces massive toxicity, while BM at the dose of 25 μg/mL is not neurotoxic, additional doses of BM in the range between 25 μg/mL and 250 μg/mL were administered to detail the dose–response for neurotoxicity. In detail, these additional doses were required to establish at which dose BM becomes neurotoxic and at which dose BM exerts full neurotoxicity. Thus, the additional range of BM doses (45 μg/mL, 65 μg/mL, 85 μg/mL, and 100 μg/mL) is no longer linear, but it allows us to dissect at which dose BM may produce neurotoxicity and the maximum neurotoxic dose. In detail, the maximal neurotoxicity of BM is obtained at the dose of 85 μg/mL, and it does not increase at the dose of 100 μg/mL. Cells were exposed to these doses of BM extracts for 72 h. Cell viability was assessed through hematoxylin and eosin (H&E) staining (Figure 1A,B and Figure S1a,b), Trypan Blue (TB) staining (Figure 1C and Figure S1c), and FJB fluorescence (Figure 2 and Figure S2). As shown in the graphs of Figure 1B,C, BM at doses ranging from 0.025 μg/mL up to 45 μg/mL, does not affect cell viability. In contrast, cell survival is reduced dose-dependently when higher doses of BM (from 65 μg/mL up to 100 μg/mL) are administered. This is shown in representative H&E pictures (Figure 1A), which report control cells and cells treated with either a low (25 μg/mL) or a high (85 μg/mL) dose of BM.

Similar results are obtained when FJB staining is carried out (representative pictures and graphs of Figure 2). Only high doses of BM, starting from 65 μg/mL, are neurotoxic and produce an increase in both the number (Figure 2B) and intensity (Figure 2C) of FJB fluorescent cells, while doses of BM below 65 μg/mL do not differ from controls (Figure 2A).

In detail, FJB-positive cells following BM at 65 μg/mL increase almost five-fold of controls, and they further increase in excess of six-fold of controls following BM doses of 85 μg/mL and 100 μg/mL (Figure 2B). Such an increase in the number of FJB-stained cells is paralleled by an increase in the mean intensity of FJB fluorescence per cell (Figure 2C). Similar results were obtained in SH-SY5Y cells (Supplementary Figure S2).

The consistency of results obtained with different methods allows the selection of the doses of BM to be challenged for neuroprotection in PC12 cells treated with METH (100 μM) or MPP^+^ (100 μM). In detail, doses of BM from 0.025 μg/mL up to 45 μg/mL were used.

### 2.2. BM Dose-Dependently Protects PC12 Cells against METH- and MPP^+^-Induced Toxicity

**Figure 3:**
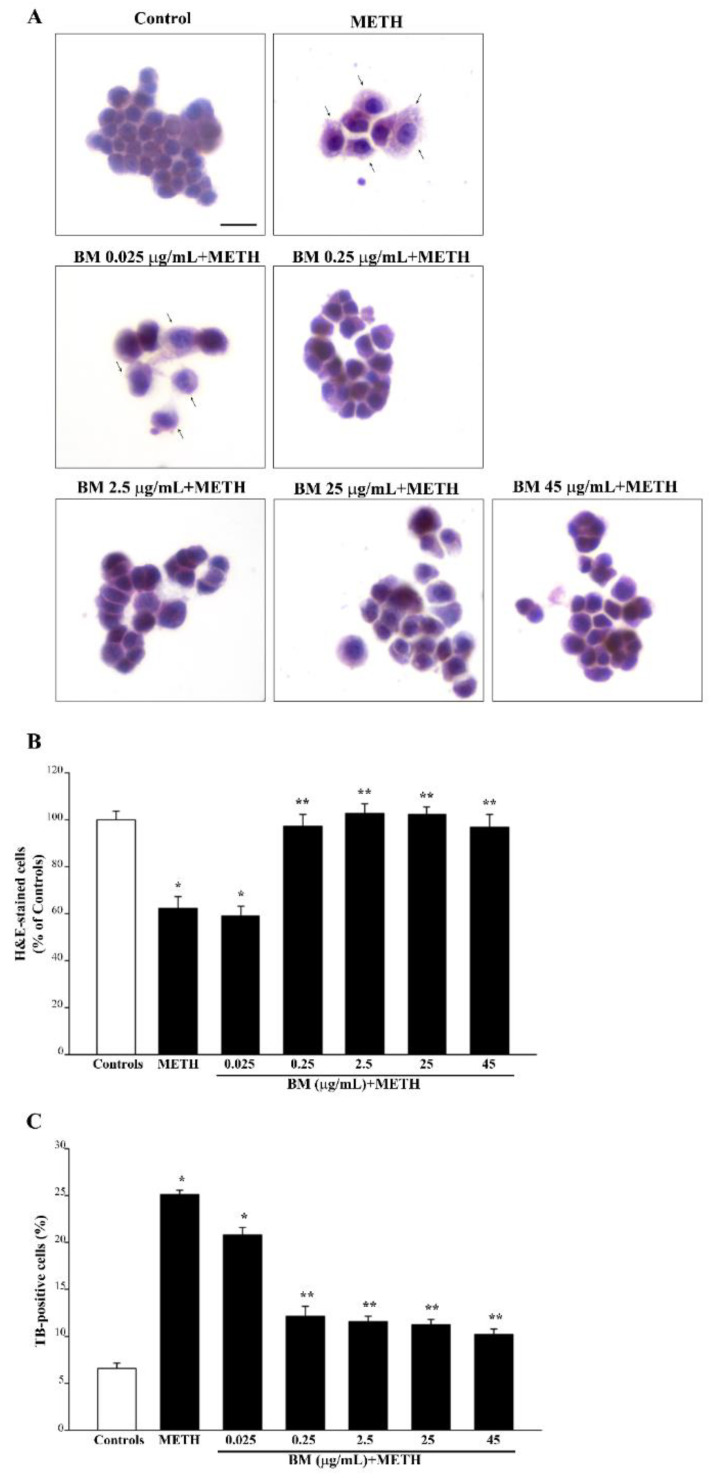
BM reduces toxicity induced by METH. (**A**) Representative H&E pictures showing marked morphological changes induced by METH (100 μM) compared with control, consisting of increased eosin staining of the cytosol and altered morphology (arrows). These alterations are reduced dose-dependently by pre-treatment with various doses of BM. Assessment of PC12 cell viability through (**B**) H&E staining, and (**C**) TB staining following exposure to METH (100 μM) alone or in combination with increasing BM doses (from 0.025 μg/mL up to 45 μg/mL). Values are given as the mean percentage ± S.E.M. of cells from three independent experiments. * *p* < 0.05 compared with controls; ** *p* < 0.05 compared with METH. Scale bar = 12 μm.

As reported in the representative pictures of Figure 3 and Figure S3, METH (100 μM) decreases cell number and alters cell morphology when examined by H&E histochemistry. In detail, spared METH-treated cells show an altered morphology and eosinophilic cytosol compared with control cells. BM (from doses of 0.025 μg/mL up to 45 μg/mL) added to METH reduces dose-dependent cell death and reverts the altered cell morphology of spared cells back to conditions described in the control (Figure 3A).

**Figure 4:**
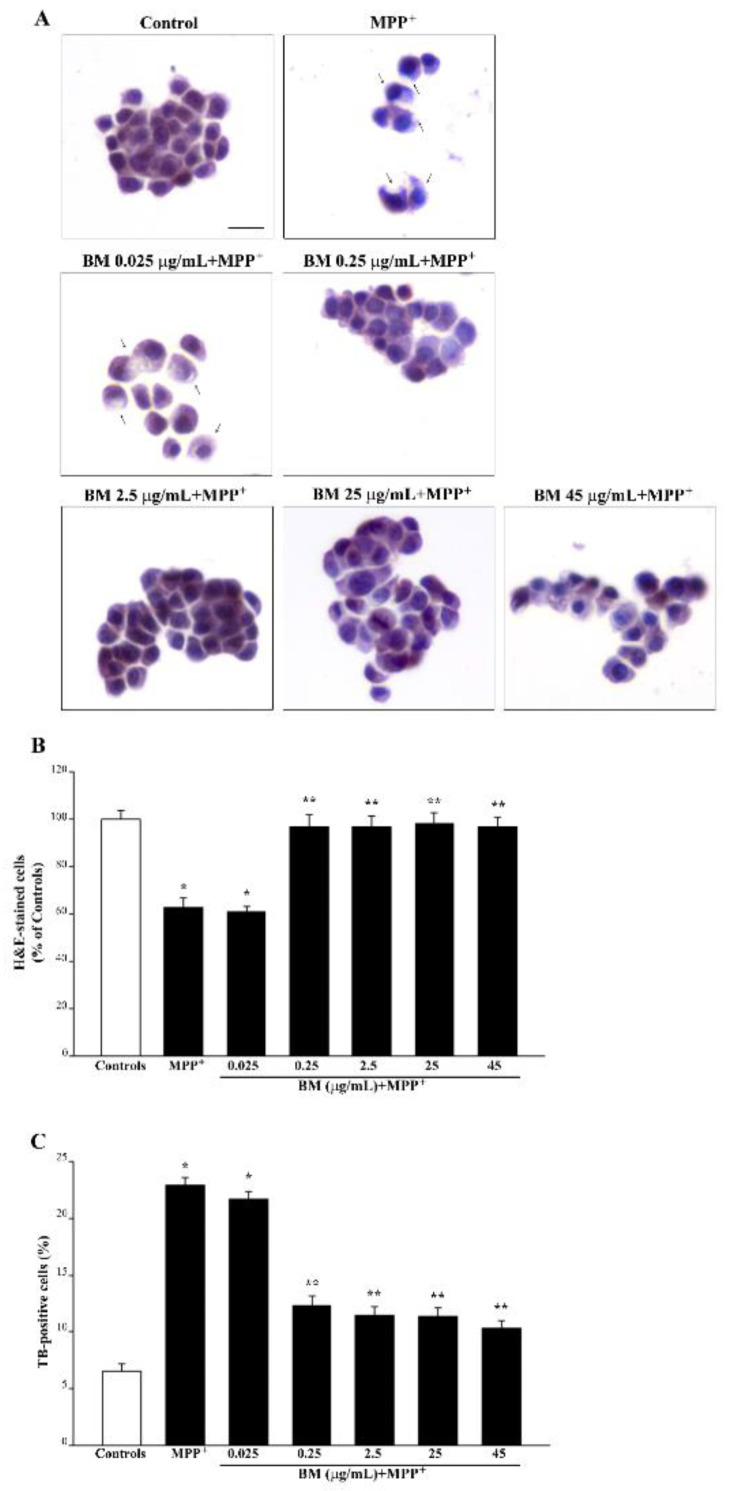
BM reduces toxicity induced by MPP^+^. (**A**) Representative H&E pictures show marked morphological changes induced by MPP^+^ (100 μM) compared with control. Note the intense hematoxylin staining and altered shape (arrows) of PC12 cells treated with MPP^+^, which is prevented by pre-treating cells with various doses of BM. Assessment of PC12 cell viability through (**B**) H&E staining, and (**C**) TB staining following exposure to MPP^+^ (100 μM) alone or in combination with increasing BM doses (from 0.025 μg/mL up to 45 μg/mL). Values are given as the mean percentage ± S.E.M. of cells from three independent experiments. * *p* < 0.05 compared with controls; ** *p* < 0.05 compared with MPP^+^. Scale bar = 12 μm.

Following MPP^+^ (100 μM), a similar amount of cell loss occurs in PC12 cells (Figure 4), while spared cells decrease their size and cytosol is intensely stained with hematoxylin (Figure 4A). Following BM, a dose-dependent decrease in cell death is evident and the intense hematoxylin staining fades away (Figure 4A). It is remarkable that METH (100 μM) and MPP^+^ (100 μM) produce a comparable amount of cell damage. In fact, as reported in Figure 3B, following METH, H&E-stained cells decrease to 62.3 ± 5.0% of controls, which is comparable to the decrease that is counted following MPP^+^ (62.9 ± 4.1% of controls, Figure 4B). In SH-SY5Y, a higher amount of cell loss was counted following MPP^+^ (46.78 ± 2.32% of controls, Supplementary Figure S4) compared with METH (56.33 ± 3.94% of controls, Supplementary Figure S3). Comparable effects for both neurotoxins are detected by counting the number of FJB cells (following METH the number of FJB cells increase in excess of four-fold of controls, similarly to MPP^+^, which increases the number of FJB cells roughly five-fold of controls). In SH-SY5Y cells, the number of FJB-stained cells increases roughly five-fold of controls following METH and about six-fold of controls following MPP^+^ (graphs of Supplementary Figure S6b and Supplementary Figure S5b, respectively).

Graphs of Figure 3 and Figure 4 indicate that BM dose-dependently protects against METH and MPP^+^ toxicity. This is consistently measured by H&E staining (Figure 3B and Figure 4B) and TB staining (Figure 3C and Figure 4C). Protection induced by BM is already evident at the dose of 0.25 μg/mL, which prevents cell loss induced both by METH and MPP^+^ (Figure 3A,B and Figure 4A,B). The very same doses of BM protect SH-SY5Y cells against METH- or MPP^+^-induced toxicity (Supplementary Figure S3 and Supplementary Figure S4, respectively).

**Figure 5:**
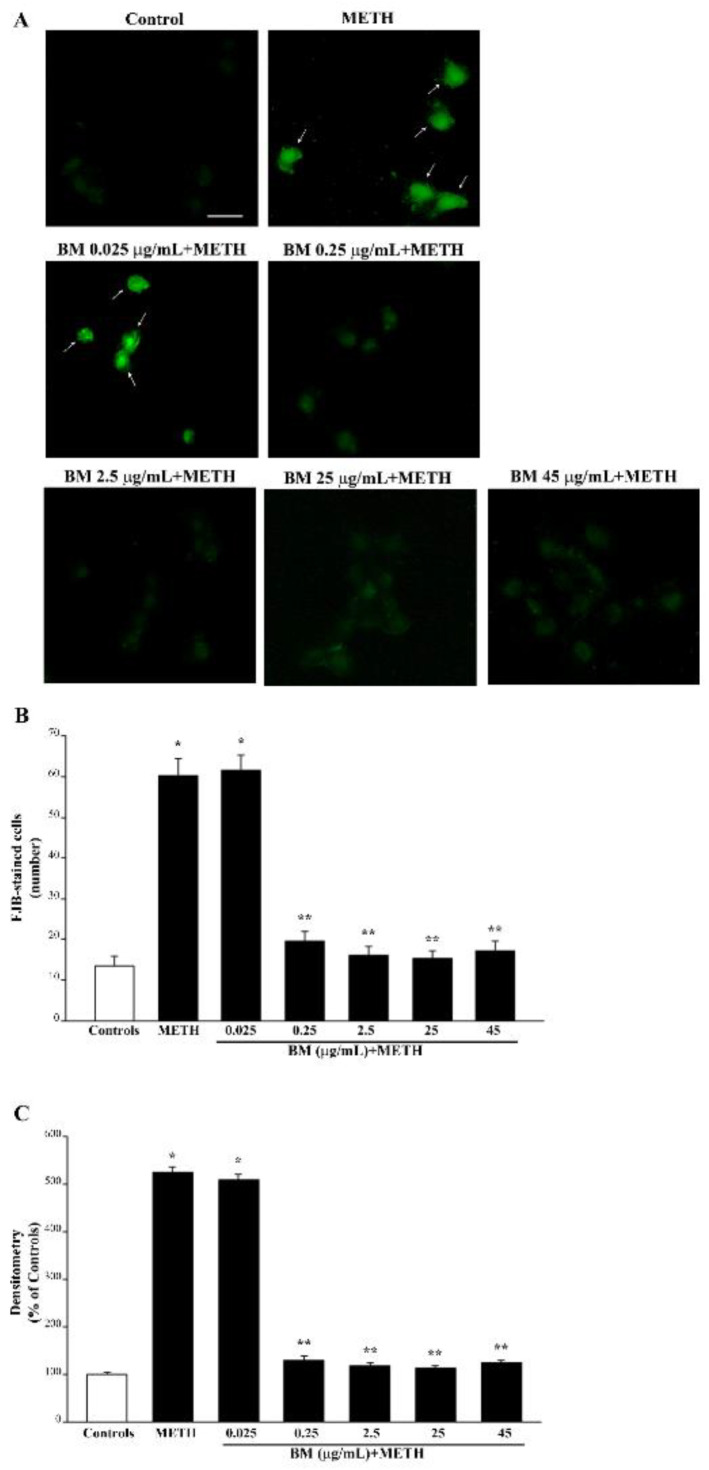
BM decreases METH-induced FJB staining. (**A**) Representative pictures of FJB-stained cells after METH (100 μM) alone or in combination with various doses of BM (from 0.025 μg/mL up to 45 μg/mL). Arrows indicate cells intensely stained with FJB. Graphs report either (**B**) the number or (**C**) the intensity of FJB fluorescent cells. Values are given as mean ± S.E.M. (**B**) or the mean percentage ± S.E.M. (assuming controls as 100%, (**C**)) of cells from three independent experiments. * *p* < 0.05 compared with controls; ** *p* < 0.05 compared with METH. Scale bar = 12 μm.

**Figure 6:**
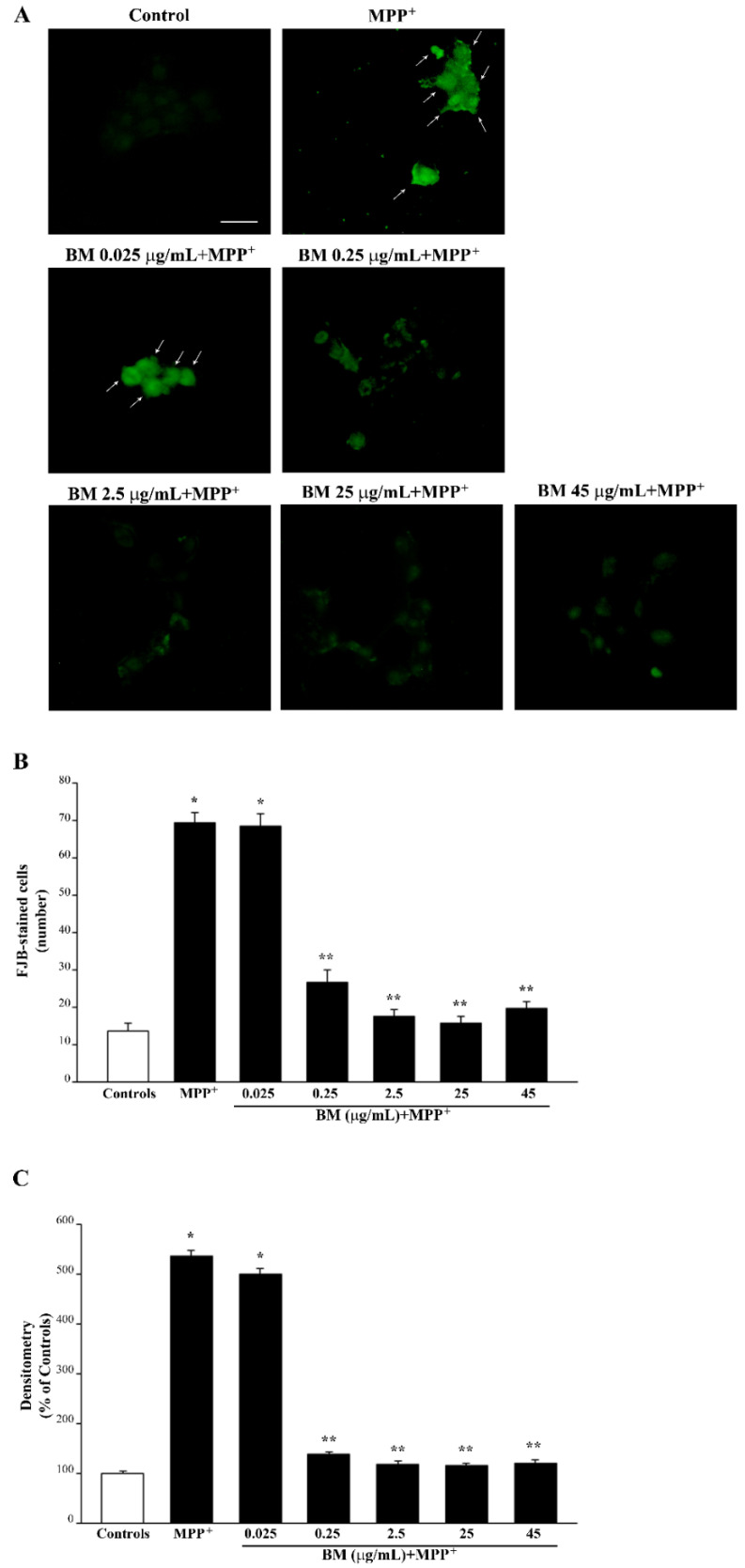
BM decreases MPP^+^-induced FJB staining of PC12 cells. (**A**) Representative pictures of FJB-stained cells after MPP^+^ (100 μM) alone or in combination with various doses of BM (from 0.025 μg/mL up to 45 μg/mL). Arrows indicate cells intensely stained with FJB. Graphs report either number (**B**) or intensity (**C**) of FJB fluorescent cells. Values are given as mean ± S.E.M. (**A**) or the mean percentage ± S.E.M. (assuming controls as 100%, (**B**)) of cells counted from three independent experiments. * *p* < 0.05 compared with controls; ** *p* < 0.05 compared with MPP^+^. Scale bar = 12 μm.

It is remarkable that, following FJB fluorescence, similar effects are obtained for METH (representative pictures Figure 5 and Figure S5) and MPP^+^ (representative pictures of Figure 6 and Figure S6). In fact, both TB and FJB mark degenerating cells, which provide a further validation of FJB as a reliable tool to assess neurodegeneration in both cell cultures. Again, by using FJB, the protective effects of BM against METH and MPP^+^ occur according to a dose–response curve (graphs of Figure 5B,C and Figure 6B,C), which is similar to that measured by using TB staining (graphs of Figure 3C and Figure 4C). The equivalence of neuroprotective doses of BM against METH and MPP^+^ toxicity suggests that BM owns analogous mechanisms of neuroprotection against both neurotoxins. In fact, these doses are steady using various staining procedures.

In detail, in the graphs of Figure 5 and Figure 6, positive FJB staining is expressed both considering the number of fluorescent cells (Figure 5B and Figure 6B) and the mean fluorescent intensity per cell (Figure 5C and Figure 6C). In these graphs, similarly to the graphs of Figure 3 and Figure 4, the same dose–response curve shows BM-induced neuroprotection. Again, this is steady along different methods and it is similar for both neurotoxins. In fact, the dose of BM 0.25 μg/mL is already fully protective, as measured following H&E (Figure 3B and Figure 4B) and TB (Figure 3C and Figure 4C) staining, which overlap with FJB staining (Figure 5B,C and Figure 6B,C). Analogous results were obtained in SH-SY5Y cells, where the dose–response curve reported in graphs of Supplementary Figures S5b,c and S6b,c is similar to that reported in Supplementary Figure S3b,c.

The efficacy of BM in protecting against METH and MPP^+^ is similar to that of rapamycin, which is reported here as a positive control since it is known to protect against both METH and MPP^+^ [59,60,61]. In fact, in the very same experimental setting, rapamycin at the dose of 100 nM provides full protection when co-administered with METH (100 μM) or MPP^+^ (100 μM), similarly to BM at the dose of 25 μg/mL (Supplementary Figures S7 and S8).

### 2.3. BM Dose-Dependently Prevents METH- and MPP^+^-Induced ROS

**Figure 7:**
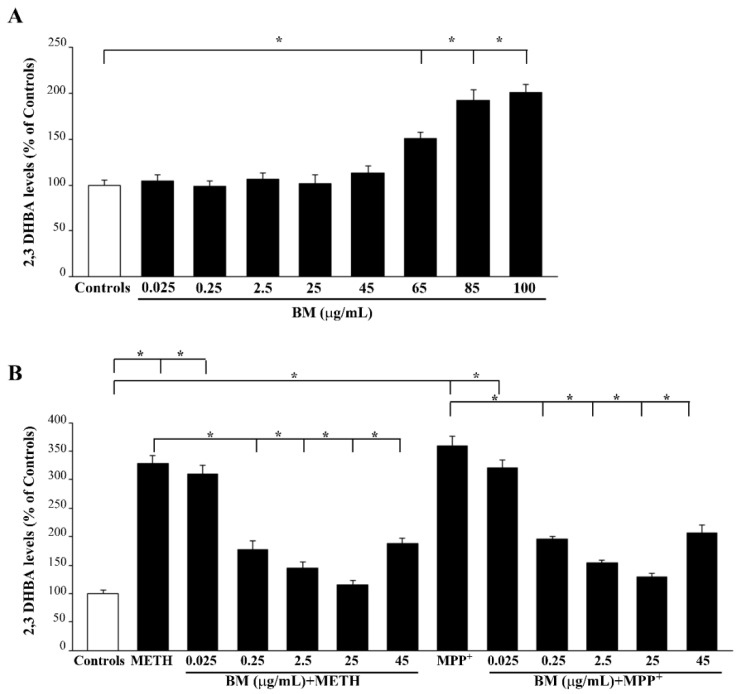
Low doses of BM decrease METH- and MPP^+^-induced ROS formation The graphs report the levels of 2,3 DHBA produced by increasing doses of BM alone (**A**) and combined treatment of BM and METH or MPP^+^ (**B**). Values are given as the mean percentage ± S.E.M. of controls (assuming controls as 100% density). Data are obtained from three independent experiments. * *p* < 0.05 compared with controls.

As reported in the graphs of Figure 7, low protective doses of BM do not alter the amount of baseline ROS formation when given alone (Figure 7A). In contrast, when given in combination with either METH or MPP^+^, small doses of BM occlude the increase in ROS, which otherwise occurs when both neurotoxins are administered alone (Figure 7B). In detail, the amount of ROS is similarly increased (350% of controls) by neurotoxic doses (100 μM) of either METH or MPP^+^. This indicates that the doses of METH and MPP^+^ being selected are equivalent in PC12 cells concerning both cell toxicity (assessed by different methods) and ROS production. In SH-SY5Y cells, compared with PC12 cells, a slightly higher vulnerability to MPP^+^ is documented. In both cell lines, low doses of BM (ranging from 0.25 μg/mL up to 45 μg/mL) dose-dependently prevent both MPP^+^- and METH-induced ROS production (Figure 7 and Figure S9). These correspond to the same doses of BM, which are effective in preventing cell toxicity in both cell cultures. This suggests that cell toxicity is tightly related to ROS production both in the case of METH and MPP^+^.

It is remarkable that when high toxic doses of BM extracts are administered alone (ranging from 65 μg/mL up to 100 μg/mL), an increase in ROS occurs (Figure 7A). However, despite a robust toxicity being produced by these high doses of BM alone, the increase in ROS being detected is much slighter (not exceeding 200% of controls) compared with that induced by MPP^+^ or METH. This suggests that the tight link between ROS production and cell death, which occurs following METH and MPP^+^, is weaker following high toxic doses of BM.

### 2.4. BM Dose-Dependently Increases Healthy (MTR-R) and Total (MTR-G) Mitochondria When Given Alone or in Combination with METH or MPP^+^

**Figure 8:**
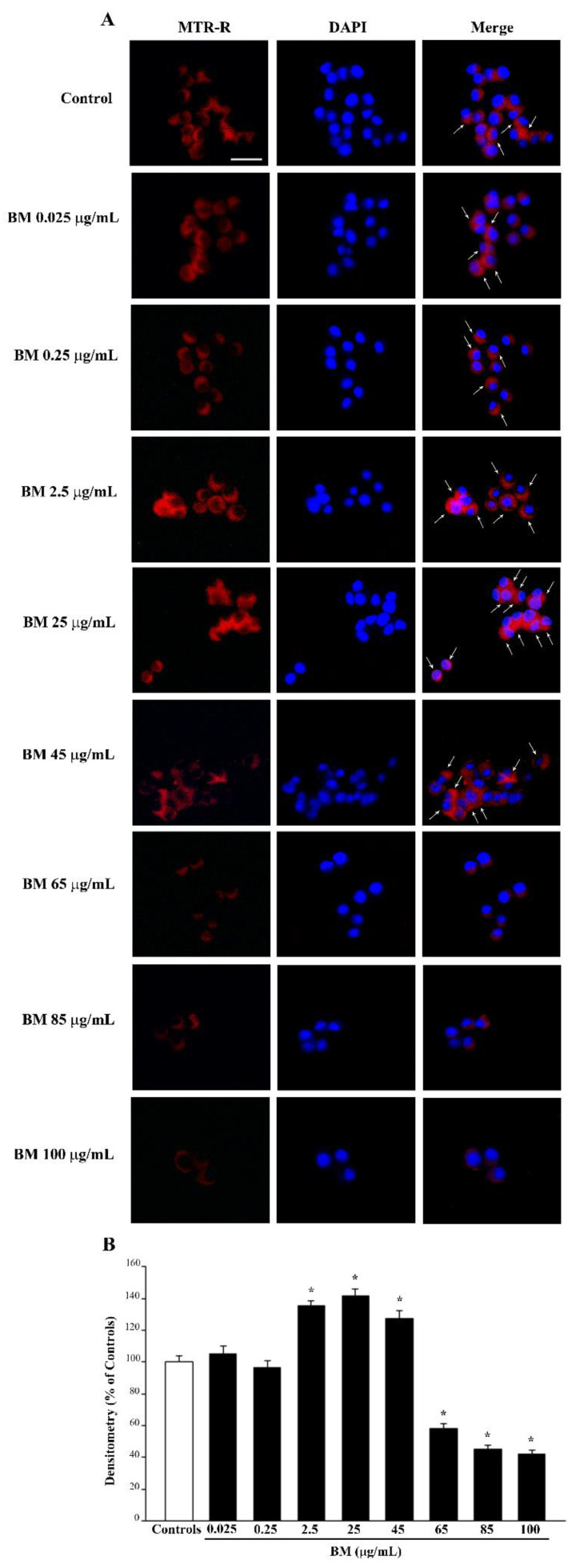
MitoTracker Red (MTR-R) fluorescence after increasing doses of BM. (**A**) Representative pictures show that low doses of BM, ranging from 0.025 μg/mL to 45 μg/mL, produce an increase in MTR-R fluorescence, which labels healthy mitochondria. In contrast, higher doses of BM (from 65 μg/mL to 100 μg/mL) decrease MTR-R fluorescence way below that of control. Arrows indicate intensely MTR-R-stained cells. Cell nuclei are stained in blue with DAPI. (**B**) The graph reports the densitometry of MTR-R fluorescence. Values are given as mean percentage *±* S.E.M. of optical density (assuming controls as 100%) from three independent experiments. * *p* < 0.05 compared with controls. Scale bar = 17 μm.

**Figure 9:**
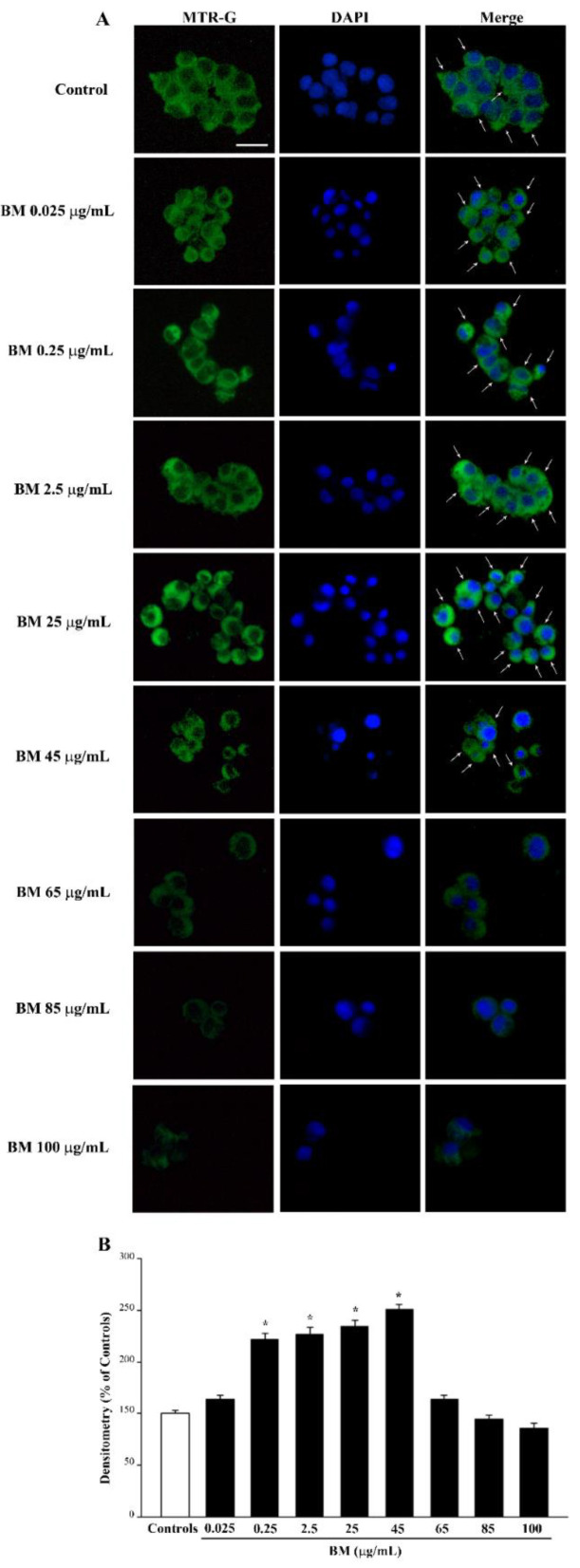
MitoTracker Green (MTR-G) fluorescence after increasing doses of BM. (**A**) Representative pictures show that MTR-G fluorescence, which labels total (both healthy and not healthy) mitochondria, is increased by low doses of BM (from 0.025 μg/mL to 45 μg/mL), while it is reduced by the highest doses of BM used in the present work (from 65 μg/mL to 100 μg/mL). Arrows indicate intensely MTR-G-stained cells. Cell nuclei are stained in blue with DAPI. (**B**) The graph reports the densitometry of MTR-G fluorescence. Values are given as mean percentage *±* S.E.M. of optical density (assuming controls as 100%) from three independent experiments. * *p* < 0.05 compared with controls. Scale bar = 17 μm.

As shown in the representative pictures of Figure 8A, neuroprotective doses of BM (from 0.25 μg/mL up to 45 μg/mL) when administered alone, increase the number of healthy mitochondria as counted in the graph of Figure 8B. Such an effect is key considering the role of these organelles in contributing to ROS formation. The increase in healthy mitochondria matches the increase in total mitochondria, as shown in the representative picture of Figure 9A and reported in the graph of Figure 9B. However, high neurotoxic doses of BM do not decrease the total mitochondria significantly compared with controls. This is due to the fact that neurotoxic doses of BM significantly decrease healthy mitochondria (Figure 8B), while the total mitochondria remain similar to controls due to the occurrence of a higher amount of damaged mitochondria (Figure 9B). This suggests that BM administered alone increases mitochondria in the cell and promotes their preservation. As expected, high, neurotoxic doses of BM decrease the number of healthy mitochondria. Similar results were obtained when the MTR-R and MTR-G stains were challenged in SH-SY5Y cells exposed to increasing doses of BM (from 00.25 μg/mL up to 100 μg/mL, Supplementary Figure S10 and Supplementary Figure S11, respectively).

**Figure 10:**
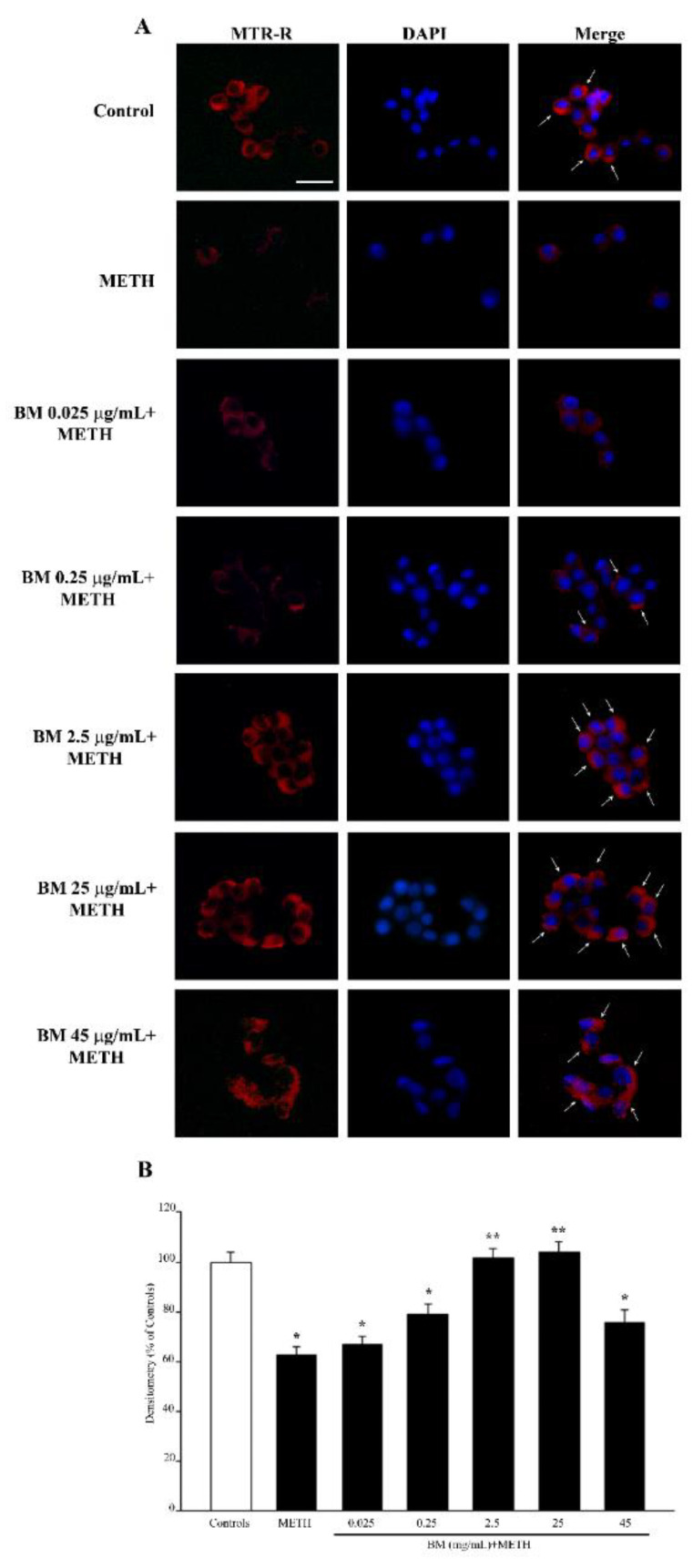
MitoTracker Red (MTR-R) fluorescence after combined treatments with BM and METH. (**A**) Representative pictures of MTR-R fluorescence show that BM counteracts the reduction in MTR-R fluorescence induced by METH. Arrows indicate intense MTR-R fluorescence. Cell nuclei are stained in blue with DAPI. (**B**) The graph reports the MTR-R fluorescence intensity. Note that protective effects of BM against METH-induced mitochondrial toxicity are evident at the doses of 2.5 μg/mL and 25 μg/mL. Values are given as mean percentage ± S.E.M. of optical density (assuming controls as 100%) from three independent experiments. * *p* < 0.05 compared with controls; ** *p* < 0.05 compared with METH. Scale bar = 17 μm.

This is mostly critical when a neurotoxin, which primarily affects mitochondria such as MPP^+^, is administered. Nonetheless, METH is also reported to affect mitochondria as a secondary effect. In fact, when tested against METH-induced mitochondrial toxicity, BM protects from the METH-induced decrease in healthy mitochondria (as shown in representative picture of Figure 10A and Figure S12a and reported in the graphs of Figure 10B and Figure S12b). It is remarkable that the dose of BM that is most effective in counteracting METH-induced mitochondrial neurotoxicity corresponds to 2.5 μg/mL, which is higher when compared with the dose (0.25 μg/mL) that is effective in providing full protection against cell death (Figure 3B and Figure 4B), cell degeneration (graphs of Figure 5 and Figure 6), and ROS production (Figure 7B). This suggests that the preservation of mitochondria by BM is not tightly related to neuroprotection against METH toxicity; rather, it shows a similar trend.

**Figure 11:**
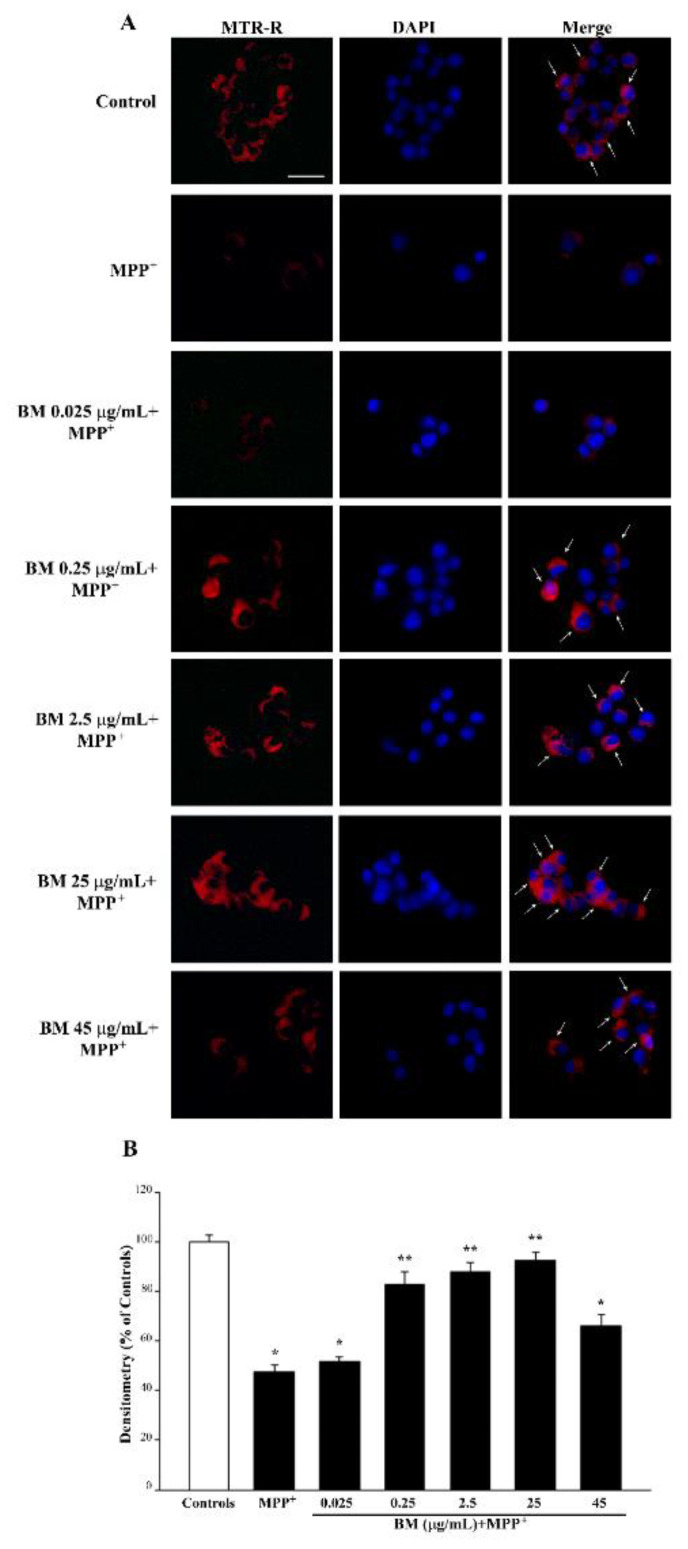
MitoTracker Red (MTR-R) fluorescent cells after combined administration of BM and MPP^+^. (**A**) Representative pictures of MTR-R fluorescence show that BM is protective against the reduction in MTR-R staining induced by MPP^+^. Arrows indicate intense MTR-R fluorescence. Cell nuclei are stained in blue with DAPI. (**B**) The graph reports the MTR-R fluorescence intensity. Note that the protective effect of BM against the MPP^+^-induced mitochondrial toxicity is already evident for the dose of 0.25 μg/mL. Values are given as the mean percentage *±* S.E.M. of optical density (assuming controls as 100%) from each experimental group from three independent experiments. * *p* < 0.05 compared with controls; ** *p* < 0.05 compared with MPP^+^. Scale bar = 17 μm.

As expected, when the primary mitochondrial neurotoxin MPP^+^ is administered, mitochondrial toxicity is more severe compared with METH (representative Figure 11A and Figure S13a and graph of Figure 11B and Figure S13b). In line with this, the most protective doses of BM against MPP^+^-induced mitochondrial are already evident at the dose of 0.25 μg/mL, which fully prevents cell loss (Figure 3B and Figure 4B), cell degeneration (graphs of Figure 5 and Figure 6), and ROS production (Figure 7B). Thus, in the case MPP^+^-treated cells, the rescue of healthy mitochondria to control levels occurs fully for the very same dose of BM, which prevents neurotoxicity. Altogether, data from MTR-R indicate that, despite both METH and MPP^+^ suppressing healthy mitochondria, the dose–response curve of BM in MPP^+^-treated cells for rescuing healthy mitochondria corresponds to the dose–response that prevents cell death, cell degeneration, and abnormal ROS production. Such a curve is shifted to the left compared with that obtained in METH-treated cells (Figure 10). This indicates that the survival of mitochondria is key to preventing MPP^+^ toxicity, while it is not so crucial for METH toxicity, where it is likely to represent a secondary phenomenon.

**Figure 12:**
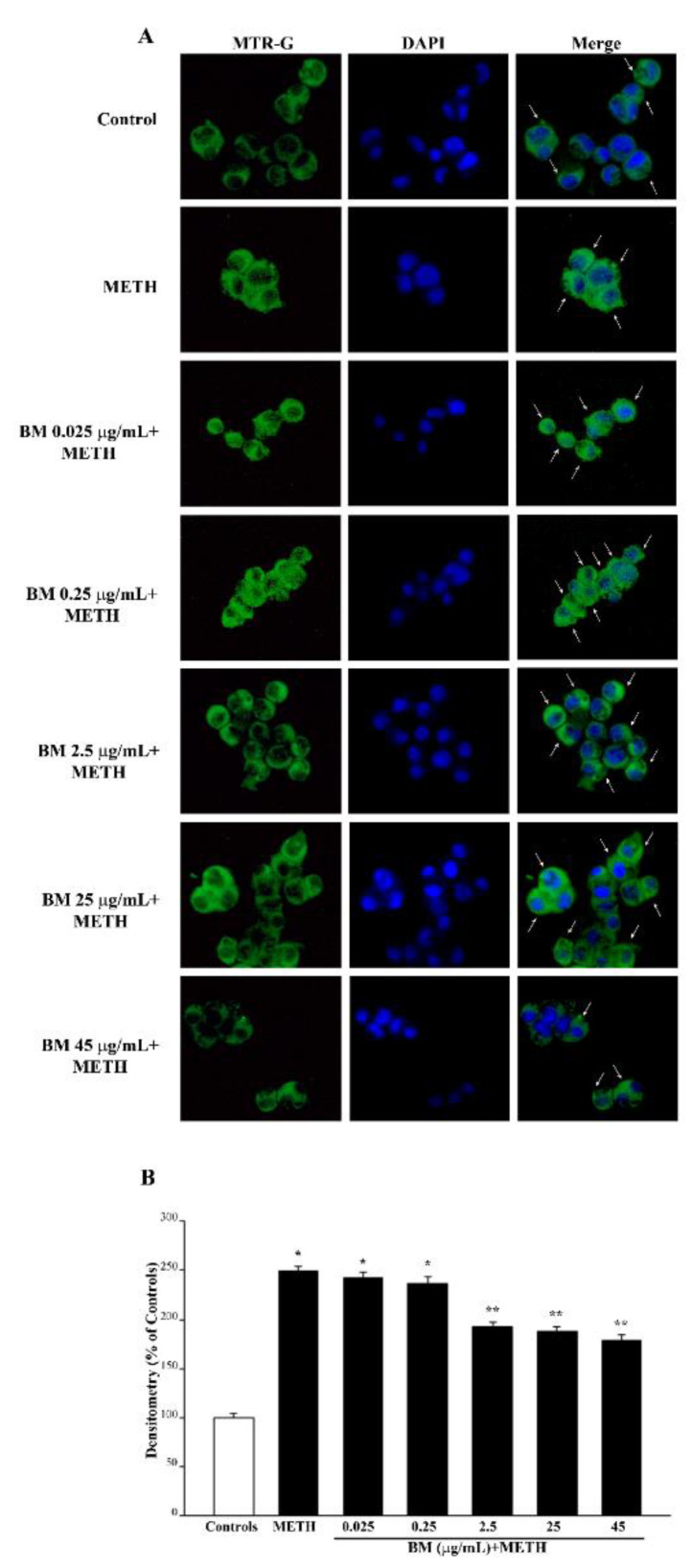
MitoTracker Green (MTR-G) fluorescent cells after combined administration of BM and METH. (**A**) Representative pictures of MTR-G fluorescence show that METH increases MTR-G fluorescence compared with control. This effect is less pronounced when METH is given in combination with BM from the dose of 2.5 μg/mL up to the dose of 45 μg/mL. This replicates what is observed under BM alone. Cell nuclei are stained in blue with DAPI. The graph in (**B**) reports MTR-G fluorescence intensity. Values are given as mean percentage ± S.E.M. of optical density (assuming controls as 100%) from three independent experiments. * *p* < 0.05 compared with controls; ** *p* < 0.05 compared with METH. Scale bar = 17 μm.

**Figure 13:**
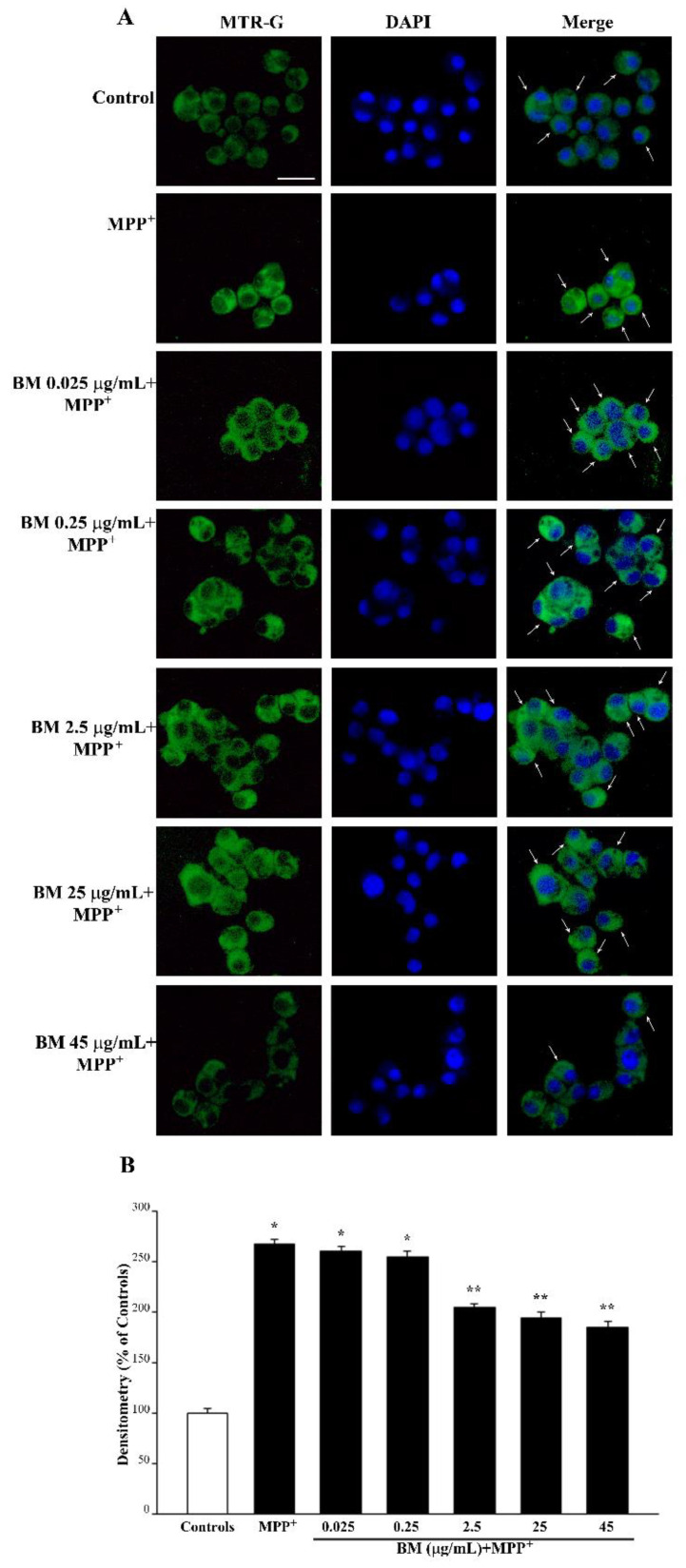
MitoTracker Green (MTR-G) fluorescent cells after combined administration of BM and MPP^+^. (**A**) Representative pictures of MTR-G fluorescence show that MPP^+^ increases MTR-G fluorescence compared with control. This effect is less pronounced when MPP^+^ is given in combination with BM from the dose of 2.5 μg/mL up to the dose of 45 μg/mL. This replicates what is observed under BM alone. Cell nuclei are stained in blue with DAPI. The graph in (**B**) reports the measures of the MTR-G fluorescence intensity. Values are given as mean percentage ± S.E.M. of optical density (assuming controls as 100%) from three independent experiments. * *p* < 0.05 compared with controls; ** *p* < 0.05 compared with MPP^+^. Scale bar = 17 μm.

In fact, as shown by MTR-G histofluorescence (representative pictures and graphs of Figure 12 and Figure S14 for METH and Figure 13 and Figure S15 for MPP^+^), BM increases the total number of mitochondria in METH- and MPP^+^-treated cells. This suggests that in order to keep steady the number of healthy mitochondria (graphs of Figure 12B and Figure S14b for METH and Figure 13B and Figure S15b for MPP^+^), other mitochondria are added to those damaged by METH or MPP^+^, which determines a marked increase in total mitochondria. The increase in total mitochondria is induced more markedly at very low doses of BM in either METH- or MPP^+^-treated cells (from 0.025 μg/mL up to 0.25 μg/mL). These data, joined with results obtained by using MTR-R, are fascinating since very low doses of BM seem to promote mitochondriogenesis, which deserves specific investigations. These data, which were obtained by using MTR-R and MTR-G histofluorescence, show the limit of a semi-quantitative assessment. Therefore, in order to better explore such an effect, in situ mitochondrial morphometry at TEM was carried out.

### 2.5. In Situ Counts of Mitochondrial Morphometry at TEM Confirms Data Obtained with MTR Histofluorescence

**Figure 14:**
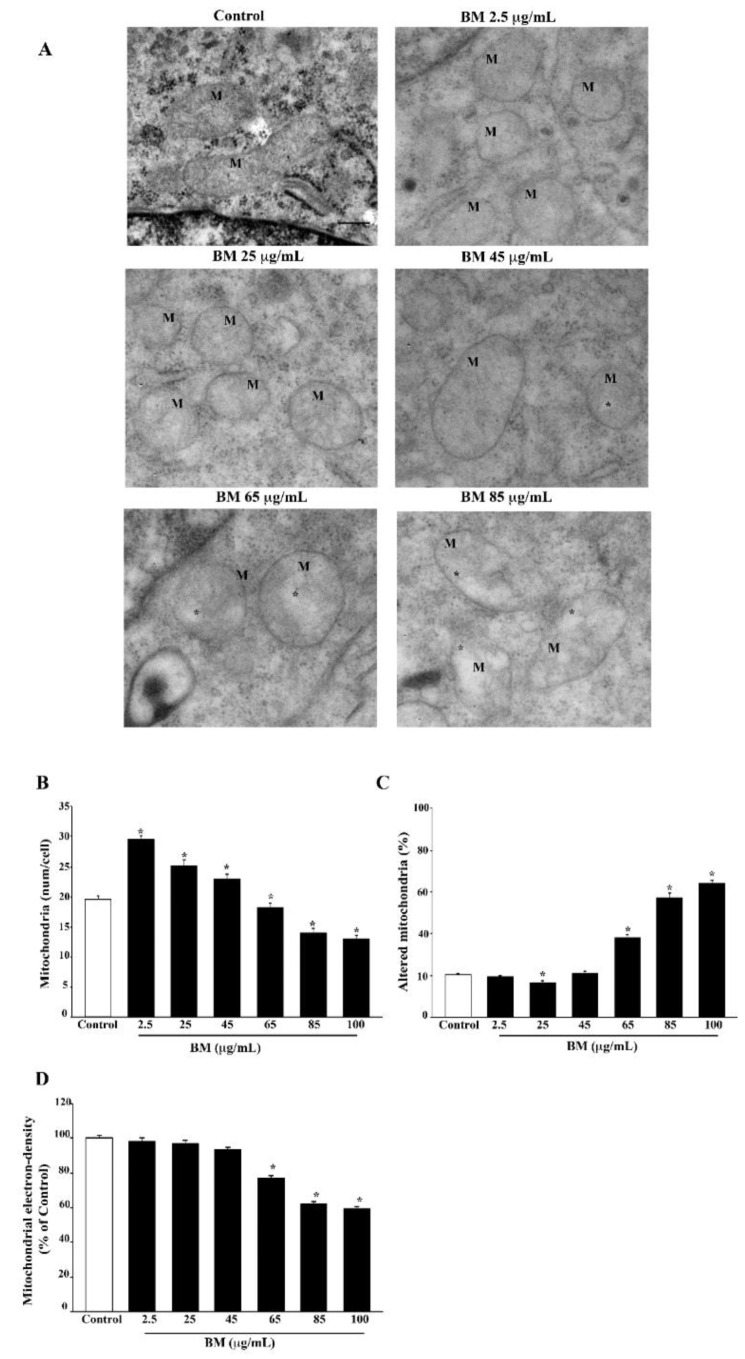
Effects of increasing doses of BM on mitochondrial ultrastructure. (**A**) Representative micrographs of mitochondria show that BM at low doses (i.e., 2.5 μg/mL and 25 μg/mL) preserves the ultrastructure of mitochondria, which appear with an electron-dense matrix and well conformed cristae. In contrast, at the highest doses used (ranging from 65 μg/mL to 85 μg/mL), BM induces mitochondrial alterations consisting of matrix dilution (*) and fragmented cristae. M = mitochondria. The graphs report the effects of increasing doses of BM on the number of total mitochondria per cell (**B**), the percentage of altered mitochondria per cell (**C**), the electron density of mitochondrial matrix (**D**). Values are given as the mean ± S.E.M. (**B**), mean percentage ± S.E.M. (**C**,**D**) of counts from three independent experiments. * *p* < 0.05 compared with controls. Scale bar = 230 nm.

Direct counts of mitochondria in situ at TEM are shown in representative pictures of Figure 14A, where the electron density of the mitochondrial matrix can be appreciated as well. In detail, BM produces a dose-dependent increase in total mitochondria and a decrease in altered mitochondria (representative pictures of Figure 14A and Figure S16a and graphs of Figure 14B–D and Figure S16b–d).

In fact, as reported in the graph of Figure 14B, the total mitochondria are increased by BM at doses ranging from 2.5 μg/mL up to 45 μg/mL. In line with MTR-G data, this effect is progressively reversed when BM is increased from 65 μg/mL up to 100 μg/mL. When counting altered mitochondria, BM increases this number, starting at doses of 65 μg/mL. It is remarkable that the dose of BM 25 μg/mL reduces the number of altered mitochondria even compared with controls (Figure 14C). The effects of BM alone on the electron density of the mitochondrial matrix are unnoticeable at low doses, while starting at the dose of 65 μg/mL BM, the mitochondrial matrix is progressively diluted to the lowest density produced by BM at the dose of 100 μg/mL (Figure 14D).

**Figure 15:**
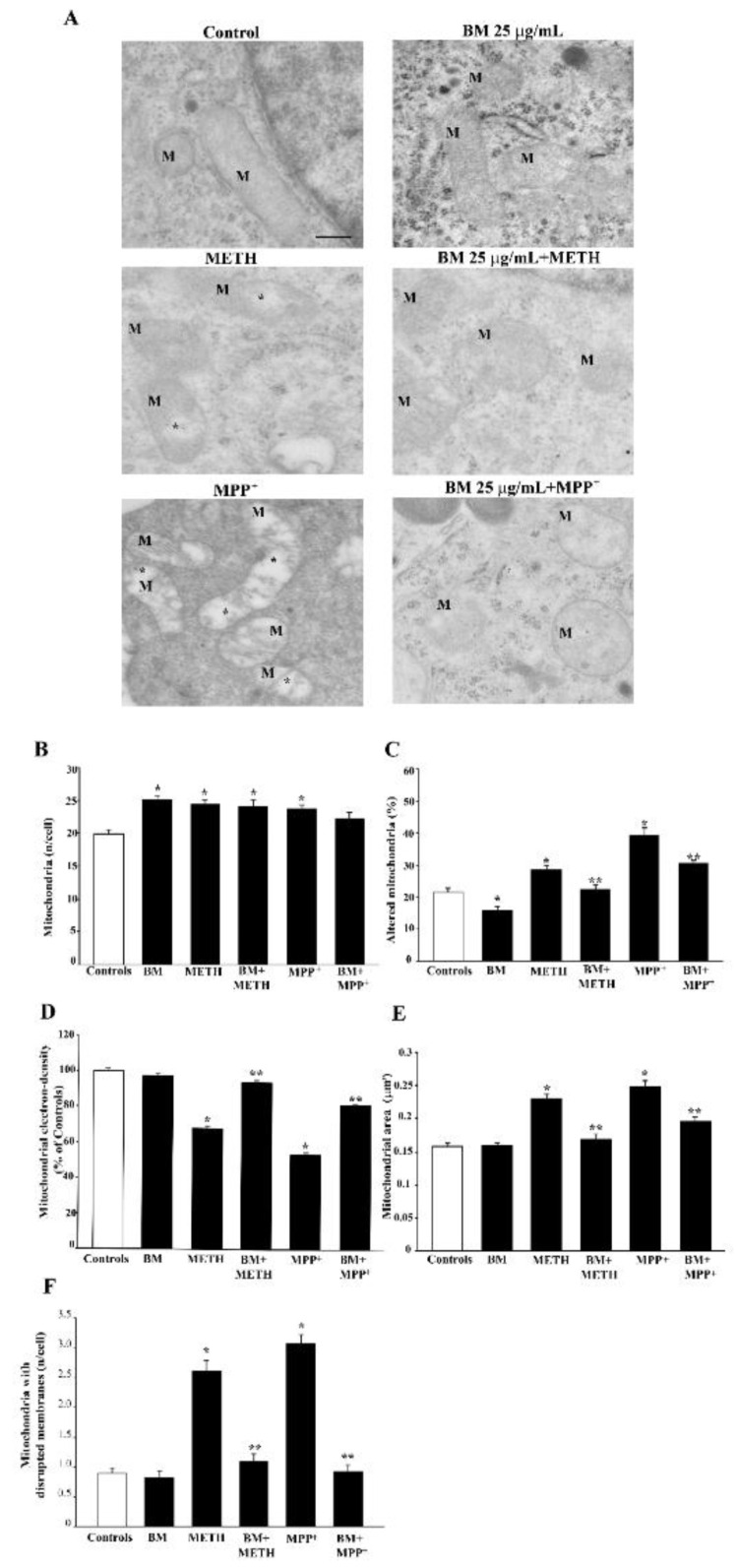
Effects of combined treatment with BM (25 μg/mL) and METH or MPP^+^ on mitochondrial ultrastructural morphometry. (**A**) Representative micrographs of mitochondria show that METH induces mitochondrial alteration, consisting of matrix dilution (*) and fragmented cristae. These mitochondrial alterations are even more severe after MPP^+^, which induces a marked loss of matrix electron density (*). Pre-treatment with BM (25 μg/mL) counteracts the mitochondrial alterations induced by both neurotoxins. Graphs report the effects of combined BM (25 μg/mL) and METH or MPP^+^ treatment on the number of total mitochondria per cell (**B**), the percentage of altered mitochondria per cell (**C**), the electron density of the mitochondrial matrix (**D**), the mitochondrial area (**E**), and the number of mitochondria with disrupted membranes (**F**). Values are given as the mean ± S.E.M. (**B**,**E**,**F**) or mean percentage ± S.E.M. (**C**,**D**) of measures from three independent experiments. * *p* < 0.05 compared with controls; ** *p* < 0.05 compared with METH or MPP^+^. M = mitochondria. Scale bar = 230 nm.

TEM analysis focused on a full protective dose of BM (25 μg/mL) to assess the protective effects of BM against METH or MPP^+^. In METH- or MPP^+^-treated cells, BM increases the number of total mitochondria and brings down to control levels the number of altered mitochondria (representative pictures of Figure 15 and graph of Figure 15B).

As shown in the graph of Figure 15C, altered mitochondria were slightly reduced by BM alone at 25 mg/mL compared with controls, while BM robustly suppressed the marked increase in altered mitochondria produced by METH, and mostly by MPP^+^ (graph of Figure 15C). A similar correction was carried out by BM concerning matrix dilution when added to either METH or MPP^+^ (graph of Figure 15D). Similarly, the mitochondrial area, which is pathologically enlarged by both MPP^+^ and METH, was re-sized by the pre-administration of BM back to control values (graph of Figure 15E). When the integrity of the inner/outer mitochondrial membrane was assessed, the number of mitochondria carrying a membrane rupture was increased similarly by MPP+ and METH, while it was fully suppressed by BM (Figure 15F). This indicates that BM counteracts neurotoxin-induced ultrastructural mitochondrial pathology. Similar results were obtained in SH-SY5Y cells (Supplementary Figure S17). This is in line with data obtained with MTR-R, while it needs to be taken into account when inferring data on the number of mitochondria by using MTR-G. In fact, a large green fluorescent area may need to be adjusted for the mitochondrial mean area before considering an actual increase in the number of mitochondria. The present ultrastructural approach allows the authentic effects on mitochondrial number and/or mitochondria area to be discerned, under the effects of various neurotoxins, with or without BM. More specifically, these data provide a direct evidence and quantification of the effects of BM on mitochondrial membranes.

## 3. Discussion

In the present study, evidence is provided that BM extracts dose-dependently protect against cell toxicity induced either by METH or MPP^+^. Remarkably, the dose–response curves for BM-induced neuroprotection against cell death are quite similar for both neurotoxins, which suggests a common mechanism of neuroprotection by BM against both neurotoxins. In fact, a significant correlation could be measured for cell death and the amount of ROS (Supplementary Figure S1), which are similarly occluded by BM. This indicates that, independently by upstream divergent mechanisms (acting on mitochondria, for MPP^+^, or DA oxidative metabolism, for METH), an increase in ROS production does occur for both neurotoxins, which is likely to contribute to destroying catecholamine cells, and this is inhibited by neuroprotective doses of BM. As expected for bioactive compounds, an excess of BM may lead to neurotoxicity. In fact, the first block of data presented in this manuscript identify which doses of BM alone may be deleterious for cell viability. In detail, while doses of BM in the range between 0.025 μg/mL and 45 μg/mL do not affect cell viability in baseline conditions, doses above 45 μg/mL up to 100 μg/mL produce noticeable detrimental effects on cell survival. These findings are worth commenting on since, so far, the potential toxicity of BM has never been detailed. This investigation was needed to better tailor the amount of BM that can be administered to achieve the maximal neuroprotection without toxicity concerns. In this way, potential BM toxicity was characterized, as we recently evidenced for other natural compounds, such as curcumin [62] and lactoferrin [63], or as shown for resveratrol [64,65].

The neuroprotective doses of BM are similar concerning either METH or MPP^+^. In addition, the amount of neuroprotection remains steady, independently of the specific procedure used to measure cell damage. This suggests that the specific procedures used in the present study, although expressing different phenomena, are equally effective in measuring cell viability. Some discrepancy may still be found due to the phenomenon that is detected by each procedure. In fact, when counting cells by using H&E, the occurrence of spared cells is relevant, independently of the occurrence of concomitant cell damage. In contrast, both TB- and FJB-positive cells express the occurrence of a dye-specific cell damage (a sort of neurodegeneration process). This means that suffering spared cells are counted as an index of damage without detecting actual cell death. In fact, the event of cell loss (which is relevant for H&E staining) does not appear for TB or FJB since no staining can be produced by lost cells. For instance, following METH (100 μM), H&E staining decreases roughly 40% compared with controls. In the same experimental conditions, TB-positive cells increase five-fold over controls and FJB-stained cells increase six-fold over controls. When BM is co-administered at the dose of 25 μg/mL, H&E staining shows a protection against METH toxicity of 100%. TB-positive cells are two-fold that of controls and FJB-positive cells are the same as controls. The neurotoxicity of MPP^+^ (100 μM) is equivalent to METH (100 μM). In fact, cell loss, which is measured with H&E, is roughly 40% compared with controls. This indicates that the doses of MPP^+^ and METH we selected in this study are truly comparable for neurotoxicity. In these conditions, TB-positive cells increase almost five-fold over controls and FJB-positive cells slightly exceed six-fold that of controls. These data are overlapping for METH and MPP^+^. Thus, in keeping with the same procedure, results are remarkably steady. This represents an internal control to validate the reliability of the methods we used and for specifically witnessing the feasibility of FJB to assess neurodegeneration even in cell culture.

The toxic effects of high doses of BM are severe (80% of cell loss compared with controls at H&E, TB-positive cells six-fold that of controls and FJB-positive cells eight-fold that of controls). This indicates that the toxicity induced by the highest dose of BM is sensed differently compared with METH and MPP^+^ toxicity by these methods. In detail, H&E doubles the amount of cell loss compared with METH or MPP^+^, while the other methods only show a slight increase. This is likely to be due to the onset of a fast neurotoxicity following high doses of BM. This is expected to produce cell death quickly (counted by cell loss with H&E) while reducing the number of degenerating dying cells (counted by TB or FJB). In fact, the neurotoxic doses of either METH or MPP^+^ produce an amount of ROS that is roughly 350% of that of controls with a cell death of 40% for both neurotoxins, while toxic doses of BM produce roughly 200% of ROS with a cell death of 80%. This confirms that toxicity induced by high doses of BM is markedly different compared with METH and MPP^+^. The protection of BM against toxicity induced by METH and or MPP^+^ is significantly correlated with the suppression of ROS formation (Supplementary Figure S18a,b). Similarly, the protection by BM against both neurotoxins occurs with a concomitant increase in healthy mitochondria, which are reduced when both neurotoxins are administered in the absence of BM. In the present study, we used two methods to assess the integrity of the mitochondrial membrane, namely MitoTracker Red (MTR-R) staining and transmission electron microscopy (TEM). We used MTR-R to stain healthy mitochondria. This fluorescent dye enters within mitochondria that possess an intact membrane potential [66]. MTR-R-stained mitochondria are expected to possess an intact membrane potential, which does reflect the occurrence of a mitochondrial membrane integrity. The staining with MTR-R still remains an indirect measurement of structural membrane integrity, and the staining of specific mitochondrial proteins does not provide a comprehensive measurement of the integrity of the inner/outer mitochondrial membranes. Therefore, this was assessed directly by TEM as reported in Figure 15F and Figure S17f. In fact, TEM is the gold standard to directly visualize the mitochondrial ultrastructure, including the mitochondrial membrane [67]. In this way, the electron density of the mitochondrial matrix, and the integrity of the mitochondrial membrane, were assessed by ultrastructural morphometry. In line with data obtained with MTR-R, both METH and MPP^+^ significantly decrease matrix electron density, while increasing disrupted mitochondrial membranes. The administration of BM fully prevents these ultrastructural changes. The assessment of specific mitochondrial proteins such as TOM20 may also be useful to study mitochondrial integrity, although the whole integrity of the mitochondrial membrane may be overlooked by measuring specific mitochondrial proteins.

The correlation between BM protection and the amount of healthy mitochondria is highly significant both for METH (Supplementary Figure S19a) and MPP^+^ (Supplementary Figure S19b). Indeed, the correlation between protection from cell death and the suppression of ROS is higher for METH compared with MPP^+^, and, conversely the correlation between protection from cell death and the amount of healthy mitochondria is higher for MPP^+^ compared with METH. These data are not surprising since METH toxicity directly depends on DA-induced ROS production (which is strongly correlated with toxicity and protection), while it may extend to mitochondria as a secondary damage. Conversely, MPP^+^ toxicity directly exerts on mitochondria. This also explains why mitochondrial preservation in the case of MPP^+^ toxicity already occurs for the lowest dose of BM (0.25 μg/mL), which is not effective in preventing METH-induced mitochondrial toxicity. This is in line with a primary role of mitochondrial alterations in altering cell survival under the effects of the neurotoxin MPP^+^. Despite METH not primarily targeting mitochondria, the protection of BM against METH toxicity is accompanied by an increase in healthy mitochondria. In fact, DA-dependent oxidative species generated directly by METH administration along with the secondary effects of METH engage mitochondria to alter their function at multiple levels in the respiratory chain [68]. This explains why METH alone produces mitochondrial alterations, which, in the present study, were documented both by light and electron microscopy.

The molecular mechanisms that produce METH toxicity are DA-dependent. In fact, METH produces a high amount of DA through the release of a massive amount of catecholamine from the vesicular storage sites [28,29,69,70,71]. In turn, DA owns strong oxidizing properties [45]. The strong oxidation promoted by DA is also related to its metabolites and partly requires the activity of the DAT [72,73]. The oxidation induced by DA leads to a defect in the clearing system that removes misfolded proteins [74].

The active neurotoxin MPP^+^ is a by-product of MPTP via monoamine oxidase type B [75,76]. MPP^+^-dependent toxicity depends on the powerful inhibition of mitochondrial complex I activity [22,25,26,27,51,52,77,78,79].

Thus, primary targets of METH and MPP^+^ in the cell differ. The way each neurotoxin produces this damage is clearly different. This explains why opposite effects may occur when specific molecules are tested to prevent METH and MPP^+^ toxicity. For instance, pre-treatment with the DA depleting agent reserpine prevents METH toxicity, while it worsens that of MPTP. Again, deletion of the protein α-synuclein from the cell provides relief from MPTP toxicity [42], while it worsens METH toxicity [43]. Similarly, monoamine oxidase inhibitors are known to exacerbate METH toxicity while protecting against MPTP toxicity [75,80]. NMDA-glutamate antagonists are protectant against METH toxicity [24,81,82,83], while they do not exert protection against MPTP [84,85,86]. Some compounds exert a dual protection. This is the case for molecules that prevent the entry of the neurotoxins within catecholamine cells. This is the case for selective blockers of presynaptic catecholamine transporters (DAT and NET). In fact, DAT and NET inhibitors prevent METH and MPP^+^ toxicity [8,9,13,18,20,21]. However, once entered in the cell, METH and MPP^+^ exert rather different effects, which are both antagonized by BM. In fact, a few studies indicate that BM owns antioxidant properties and protects mitochondria from oxidative damage [53,87,88,89,90]. This is why BM was previously used to protect from MPTP/MPP^+^ toxicity in animal models of parkinsonism [54,56]. In these studies, pre-treatment with BM protected against MPTP-induced nigrostriatal DA loss. This BM-induced neuroprotection was achieved by a robust antioxidative and antiapoptotic effect [55,56,91] and by increasing the expression of neurogenic genes [54]. Interestingly, a recent study has shown that BM, and bacopaside-XII, inhibit monoamine oxidase type B (MAO-B), the enzyme that converts MPTP into its active metabolite MPP^+^ [56].

In the present study, we chose to use undifferentiated PC12 cells due to their well-characterized cell profile, which was previously investigated [92]. In this study, a specific comparison between the PC12 cell body and the DA axon terminal was carried out. This previous study shows that PC12 cell bodies possess a vulnerability to neurotoxins, which is similar to DA axon terminals. This correlates with a marked polarization of synaptic vesicles to the active release zones of the plasma membrane [92]. This is reminiscent of axon terminals, while it does not occur in neuronal cell bodies in vivo. Remarkably, when we extended our investigation to the neuroblastoma cell line SH-SY5Y, which possesses similar ultrastructural features concerning vesicle polarization, similar results were obtained.

Neuroprotection of BM against MPTP-induced neurotoxicity extends to other in vivo models, such as zebrafish [93] and *Caenorhabditis elegans* [57]. In zebrafish, BM-coated nanoparticles promote the rescue of mitochondrial complex I activity, which is suppressed following MPTP [93].

The present study represents the first investigation on the effects of BM in METH-induced neurotoxicity. To our knowledge, there is no previous study that has investigated such an effect, either in vitro or in vivo. In line with this, some previous studies documented that BM suppresses hyperlocomotion [94,95] and stereotypies [96] induced by amphetamine in vivo.

The present findings show that BM protects from experimental parkinsonism. This adds on to other natural compounds that exert neuroprotective effects against MPP^+^-induced neurotoxicity through antioxidant and/or antiinflammatory activity [97,98], as well as by protecting from MPP^+^-induced mitochondrial damage [99]. A very recent study documented that a natural alkaloid derivative is able to prevent MPP^+^-induced DA cell loss both in vitro and in vivo by inhibiting apoptosis and by stimulating autophagy [100].

In the present study, BM protection against MPP^+^ was better detailed in the relationship with mitochondrial alterations and ROS production, which are both attenuated by BM. This deserves dedicated investigations encompassing specific proteins regulating mitochondrial fission, mitophagy, and mitochondriogenesis. This may be addressed in studies aimed at analyzing a potential role for BM to remove altered mitochondria via induction of the autophagy machinery [100,101].

## 4. Materials and Methods

### 4.1. Cell Cultures

Pheochromocytoma PC12 cell line is grown in house, and originally it was purchased from IRCCS San Martino Institute (Genova, Italy). Cells were grown in RPMI 1640 medium (Sigma-Aldrich, St. Louis, MO, USA), supplemented with horse serum (HS, Sigma-Aldrich), fetal bovine serum (FBS, Sigma-Aldrich), and antibiotics.

SH-SY5Y cells lines were obtained from the American Type Culture Collection (ATCC, Rockville, MD, USA) and were grown in 50% minimal essential medium (Sigma-Aldrich) and 50% F12 nutrient medium supplemented with 10% heat-inactivated fetal bovine serum and 2 mML glutamine.

Cells were kept in wet atmosphere with 5% CO_2_ at 37 °C. Experiments were performed in the log-phase of growth, at 70% confluence [102,103].

Cells were seeded and incubated at 37 °C in 5% CO_2_ for 24 h. This brought up the number of cells used to roughly 10^6^ per each experimental group.

### 4.2. Cell Treatments and Experimental Design

*Bacopa monnieri* extract, which contains 10% of bacosides, was kindly provided by Aliveda (Aliveda, Crespina Lorenzana, Pisa, Italy). A stock solution of BM was prepared by dissolving 1 mg of BM powder in 1 mL of warmed (37 °C) culture medium containing 10% of dimethyl sulfoxide (DMSO; Sigma-Aldrich).

When dissolved in phosphate buffer (pH 6.8–9.0), bacosides have been shown to be stable for at least 5 days [104]. The culture medium possesses a pH = 7–7.6, which is within the range of pH values that guarantee the stability of bacosides. Aliquots of the BM stock solution containing 10% DMSO were added to the cell culture to obtain the final treatment solutions, where DMSO was further diluted, reaching a final concentration of roughly 1%. Remarkably, this concentration of DMSO is documented to improve the cell permeability to BM [105]. The use of DMSO, due to its powerful solvent properties for organic and inorganic chemicals, fosters the solubility of BM, and it is commonly used to prepare both storage solutions, where BM extracts are dissolved in DMSO alone [106,107], and treatment solution of BM, which contains variable concentrations of DMSO, ranging from 0.1% up to 2% [106,107,108]. In our experiments, the concentration of DMSO in the final treatment solutions increased when increased volumes of the BM stock solution were used. Therefore, the highest BM dose (100 μg/mL) contains the highest DMSO concentration, corresponding to 1%. To evaluate whether this DMSO concentration affects cell viability, each BM (and DMSO) concentration was controlled, exposing PC12 cells for 72 h to a culture medium containing from 2.5 × 10^−4^ up to 1% DMSO. In this range of doses of DMSO, no effects on cell viability were assessed compared with cells exposed to culture medium.

A stock solution of METH (kindly gifted by Forensic Medicine, University of Pisa), 10 mM, was prepared by dissolving 2.3 mg of METH directly in 1 mL of culture medium, while a stock solution of 10 mM MPP^+^ (Sigma-Aldrich) was obtained by dissolving 3.0 mg of MPP^+^ in 1 mL of culture medium. The final treatment solutions were obtained by diluting appropriate aliquots of each stock solution within the culture medium.

In a first set of experiments, the safety of BM was evaluated by administering increasing doses of BM, ranging from 0.025 μg/mL up to 100 μg/mL (i.e., 0.025 μg/mL, 0.25 μg/mL, 2.5 μg/mL, 25 μg/mL, 45 μg/mL, 65 μg/mL, 85 μg/mL, and 100 μg/mL) for 72 h. Analysis of cell viability, carried out by Trypan Blue (TB), hematoxylin and eosin (H&E), and Fluoro Jade B (FJB) staining, allowed selection of the doses of BM that are not toxic for PC12 cells. These BM doses, corresponding to 0.025 μg/mL, 0.25 μg/mL, 2.5 μg/mL, 25 μg/mL, and 45 μg/mL, were challenged in order to evaluate their potential neuroprotective activity against two different neurotoxins, METH and MPP^+^. To this purpose, no toxic doses of BM were administered in combination with a frankly neurotoxic dose of either METH or MPP^+^. Co-treatments with BM and METH or MPP+ were carried out by pre-administering BM to the cell culture 2 h before the neurotoxins METH or MPP^+^. This allowed investigation of the effectiveness of BM in preventing the neurotoxicity induced by METH or MPP^+^. The dose of METH or MPP+ used in this study were selected in pilot experiments, in order to obtain for each neurotoxin roughly 30% of cell death. This amount of cell death was produced by a METH dose of 100 μM and a MPP+ dose of 100 μM. In these combined BM + METH and BM + MPP^+^ treatments, the neuroprotective effect of BM against METH- or MPP^+^-induced toxicity was evaluated using H&E, TB, and FJB staining.

Experiments with rapamycin were carried out by administering rapamycin 2 h before METH. The treatment solution (100 nM) was prepared, adding to the cell culture an adequate volume of a stock solution of rapamycin, which was obtained by dissolving rapamycin 1 mM in the culture medium containing 10% DMSO.

In a second set of experiments, measurement of ROS was carried out in order to evaluate the involvement of oxidative stress in the cell death induced either by each neurotoxin or high doses of BM alone (≥65 μg/mL), and to test the hypothesis that the neuroprotective effect of BM was associated with a reduction of oxidative stress.

Finally, since mitochondria represent the cell organelle, which is a primary (for MPP^+^) or secondary (for METH) target of neurotoxins used in the present study, mitochondrial integrity was analyzed by light and electron microscopy. In detail, the amount of total and healthy mitochondria was measured at light microscopy through MitoTracker Green (MTR-G) and MitoTracker Red (MTR-R) fluorescence, respectively. This was carried out both following increasing doses of BM alone and combined treatments with BM and METH or MPP^+^.

Finally, the effects of the various treatments on mitochondrial morphology were analyzed using transmission electron microscopy (TEM). In detail, ultrastructural morphometry was carried out in order to measure: (i) the amount of total mitochondria; (ii) the amount of altered mitochondria; (iii) the electron density of the mitochondrial matrix; (iv) the mitochondrial area.

### 4.3. Trypan Blue (TB) Staining

To assess the percentage of dying cells, TB staining was carried out. Twenty-four h before treatment, PC12 or SH-SY5Y cells were seeded at a density of 10^4^ cells/well and placed within 24-well plates in 1 mL of culture medium. After treatment, cells were collected and centrifuged at 800× *g* for 5 min, the cell pellet was suspended in 0.5 mL of the original culture medium, and 25 μL of cell suspension was incubated for 10 min in a solution of 1% TB (62.5 μL) and PBS (37.5 μL). Ten μL aliquot of this solution was analyzed using a Bürker chamber under the Olympus CKX 41 inverted microscope (Olympus Corporation, Tokyo, Japan). Viable and nonviable cells were counted, and cell death was expressed as the mean percentage ± S.E.M. of TB-positive cells out of the total cells. Data represent the means of three chamber counts, which were replicated for three independent experiments.

### 4.4. Hematoxylin and Eosin (H&E) Histochemistry

For H&E staining, 5 × 10^4^ PC12 or SH-SY5Y cells were seeded on poly-lysine coverslips and placed in 24-well plates in a final volume of 1 mL/well.

Cells were fixed using 4% paraformaldehyde in phosphate-buffered saline (PBS) solution for 15 min and subsequently washed in PBS and immersed in hematoxylin solution (Sigma-Aldrich) for some min. The slides were washed out to stop hematoxylin staining and immediately plunged within the eosin solution (Sigma-Aldrich). After repeated washing to remove the excess of dye, cells were dehydrated in increasing alcohol solutions, clarified in xylene, covered with DPX mounting medium (Sigma-Aldrich), and finally observed under a Nikon Eclipse 80i light microscope (Nikon, Tokyo, Japan).

Cell count was performed by light microscopy at 20× magnification; the number of stained cells detectable after each specific treatment was counted and expressed as the mean percentage ± SEM of the control group. Data were obtained in three independent experiments.

### 4.5. Fluoro Jade B (FJB) Histofluorescence

Fluoro Jade B (FJB) staining [109] was carried out in 5 × 10^4^ PC12 or SH-SY5Y cells, which were seeded on poly-lysine coverslips and placed in 24-well plates in a final volume of 1 mL/well.

After washing in PBS, cells were fixed with paraformaldehyde 4% for 5 min, incubated with 0.06% potassium permanganate for 10 min at room temperature, and washed again in distilled water. Then, cells were incubated with 0.0004% FJB (Merck Millipore, Billerica, MA, USA) solution (consisting in 0.01% FJB in acetic acid) at room temperature for 20 min and cover slipped with mounting medium. FJB-positive cells were analyzed by Nikon Eclipse 80i light microscopy (Nikon, Tokyo, Japan), equipped with a florescence lamp and a digital camera connected to the NIS Elements software for image analysis (Nikon, Tokyo, Japan). For each experimental group, the count of FJB-positive cells was carried out at 20× magnification. Values were expressed as the mean number ± SEM for each experimental group. The intensity of the fluorescent signaling was measured under florescence microscopy using the software Image J (NIH, USA, Version 1.8.0_172), and values are expressed as the mean percentage ± S.E.M. of optical density (assuming controls as 100%) measured in *n* = 90 cells/group. All data refer to three independent experiments.

### 4.6. Measurement of Reactive Oxygen Species (ROS)

Formation of ROS was examined by monitoring the amount of the by-product 2,3-dihydroxybenzoic acid (2,3-DHBA), which is derived from the reaction of salicylate with hydroxyl radicals occurring in the supernatant. Thus, the amount of 2,3-DHBA provides an indirect measure of the extracellular ROS levels [110]. To this purpose, 0.5 M of salicylate was added to the culture medium for 1 h at room temperature in the dark. Then, PC12 cells and culture medium were picked up from the well and they were centrifuged at 1000× *g* for 5 min in order to obtain the supernatant. Twenty μL of supernatant was used to quantify the amount of 2,3-DHBA by HPLC with electrochemical detection Coulochem II electrochemical detector (ESA, Inc., Chelmsford, MA, USA), as previously published [31,111]. The amount of 2,3-DHBA was expressed as the mean percentage ± S.E.M. of baseline values which were measured in three independent experiments.

### 4.7. Mitochondrial Labelling

To stain mitochondria in living cells, 5 × 10^4^ PC12 or SH-SY5Y cells were grown in 24-well plates containing 1 mL/well of culture medium. At the end of the treatments, the medium was removed, and cells were incubated in a solution of MitoTracker Red (MTR-R, Thermo-Fisher Scientific, Waltham, MA, USA) or MitoTracker Green (MTR-G, Thermo-Fisher Scientific) at 500 nM in a serum-free culture medium for 45 min, at 37 °C and 5% CO_2_. MTR-R allows healthy mitochondria to be revealed, whereas MTR-G labels total (both healthy and unhealthy) cell mitochondria [112,113,114]. Cell nuclei were stained with the fluorescent dye DAPI (Sigma-Aldrich). At the end of incubation, MTR-R or MTR-G solution was removed, fresh pre-warmed medium was added, and cells were immediately analyzed at fluorescence microscopy (Nikon). The optical density was measured under fluorescence microscope using Image J software (NIH, USA, Version 1.8.0_172). Values are given as the mean percentage ± S.E.M. of the optical density of *n* = 90 cell/group (assuming controls as 100%), which were obtained in three independent experiments.

### 4.8. Transmission Electron Microscopy (TEM)

For transmission electron microscopy (TEM), 1 × 10^6^ cells were seeded in culture dishes in a final volume of 5 mL. For PC12 cells, after centrifugation at 1000× *g* for 5 min, the cell pellet was rinsed in PBS and fixed for 90 min at 4 °C in a fixing solution containing 2.0% paraformaldehyde and 0.1% glutaraldehyde in 0.1 M PBS (pH 7.4). For SH-SY5Y cells, after removing culture medium, the fixing solution (2.0% paraformaldehyde/0.1% glutaraldehyde in 0.1 M PBS pH 7.4) was added to cell culture for 90 min at 4 °C. Cells were gently scraped from the plate and centrifuged at 10,000 rpm for 10 min to obtain the cell pellet.

After washing in PBS (0.1 M), both PC12 and SH-SY5Y specimens were post-fixed in 1% osmium tetroxide (OsO_4_) for 1 h, at 4 °C, and they were dehydrated in increasing ethanol solutions (30%, 50%, 70%, 90%, and 95% for 5 min, and 100% for 60 min) to be embedded in epoxy resin.

Ultrathin sections (90 nm thick) were cut at ultra-microtome (Leica Microsystems, Wetzlar, Germany), counterstained with a saturated solution of uranyl acetate and lead citrate, dissolved in distilled water, and they were finally examined using a JEOL JEM SX100 transmission electron microscope (JEOL, Tokyo, Japan).

### 4.9. Ultrastructural Morphometry of Mitochondria

Grids containing non-serial ultrathin sections were examined at 6000× magnification. A number of at least 30 cells per each experimental group were examined in order to count both total number and altered number of mitochondria per cell. Starting from a grid square corner, the whole sectioned pellet was scanned in equally spaced parallel sweeps across the specimens. Mitochondria were identified for their shape and structure. This latter consists of a typical double-membrane, which limits an intermembrane space; the area internal to the inner membrane features a sort of labyrinth system, where a homogeneous matrix and quite regularly intermingled crests (cristae) are evident. This general ultrastructural morphology of the mitochondria, though standardized, may vary at high magnification depending on cell metabolism and pathology [67,114]. The number of mitochondria with disrupted inner/outer membrane structures was calculated following both MPP+ and METH. This was replicated both in PC12 and SH-SY5Y cells, where the protective effects of BM were assessed.

Moreover, electron density of the mitochondrial matrix and mitochondrial area were measured by using ImageJ software (NIH, USA, Version 1.8.0_172). In detail, mitochondrial electron density was normalized by measuring the raw electron density of mitochondria compared with the mean raw electron density of the cytoplasm. Measurement of the mitochondrial area was carried out by using the tool “freehand selection” of Image J and reported as μm^2^.

### 4.10. Statistical Analysis

For cell viability experiments, the number of H&E-stained cells was expressed as the mean percentage ± S.E.M. of cells counted in three independent experiments (assuming controls as 100%).

TB-positive cells were expressed as the mean percentage ± S.E.M. of TB-positive cells out of the total cells, counted in three independent experiments.

The number of FJB-positive cells were expressed as the mean ± S.E.M. of FJB-positive cells counted in three independent experiments.

For FJB, MTR-R, and MTR-G, the optical density of each sample was calculated. Values were expressed as the mean percentage ± S.E.M. of the fluorescent densitometry with respect to the control, which was measured in *n* = 90 cells/group in three independent experiments.

The amount of 2,3-DHBA was expressed as the mean percentage ± S.E.M. of baseline values, which were measured in three independent experiments.

For ultrastructural morphometry, the number of total and altered mitochondria per cell were expressed as the mean or the mean percentage ± S.E.M. per cell, respectively (*n* = 30 cells per group).

The mitochondrial electron density and the mitochondrial area were expressed as the mean percentage± S.E.M. and the mean± S.E.M. respectively, measured in 150 (PC12) or 100 (SH-SY5Y) mitochondria per experimental group. The number of mitochondria featuring a rupture of inner/outer mitochondrial membrane was expressed as the mean± S.E.M. measured in 50 cells per experimental group.

Comparisons among different groups were carried out by one-way analysis of variance (ANOVA), followed by Scheffé’s post hoc analysis. The null hypothesis (H_0_) was rejected for *p* ≤ 0.05.

## Data Availability

The data that supports the findings of this study are available from the corresponding author upon reasonable request.

## Supplementary Materials

The following supporting information can be downloaded at: https://www.mdpi.com/article/10.3390/molecules27165204/s1.
