# Supplementary material for: Bacopa Protects against Neurotoxicity Induced by MPP+ and Methamphetamine
Source: Molecules. 2022 Aug 15;27(16):5204. doi: 10.3390/molecules27165204 (PMC9414486; doi:10.3390/molecules27165204)
Supplement: Supplementary file 1 [file molecules-27-05204-s001.zip › molecules-1804863-supplementary.pdf]

Supplementary Figures

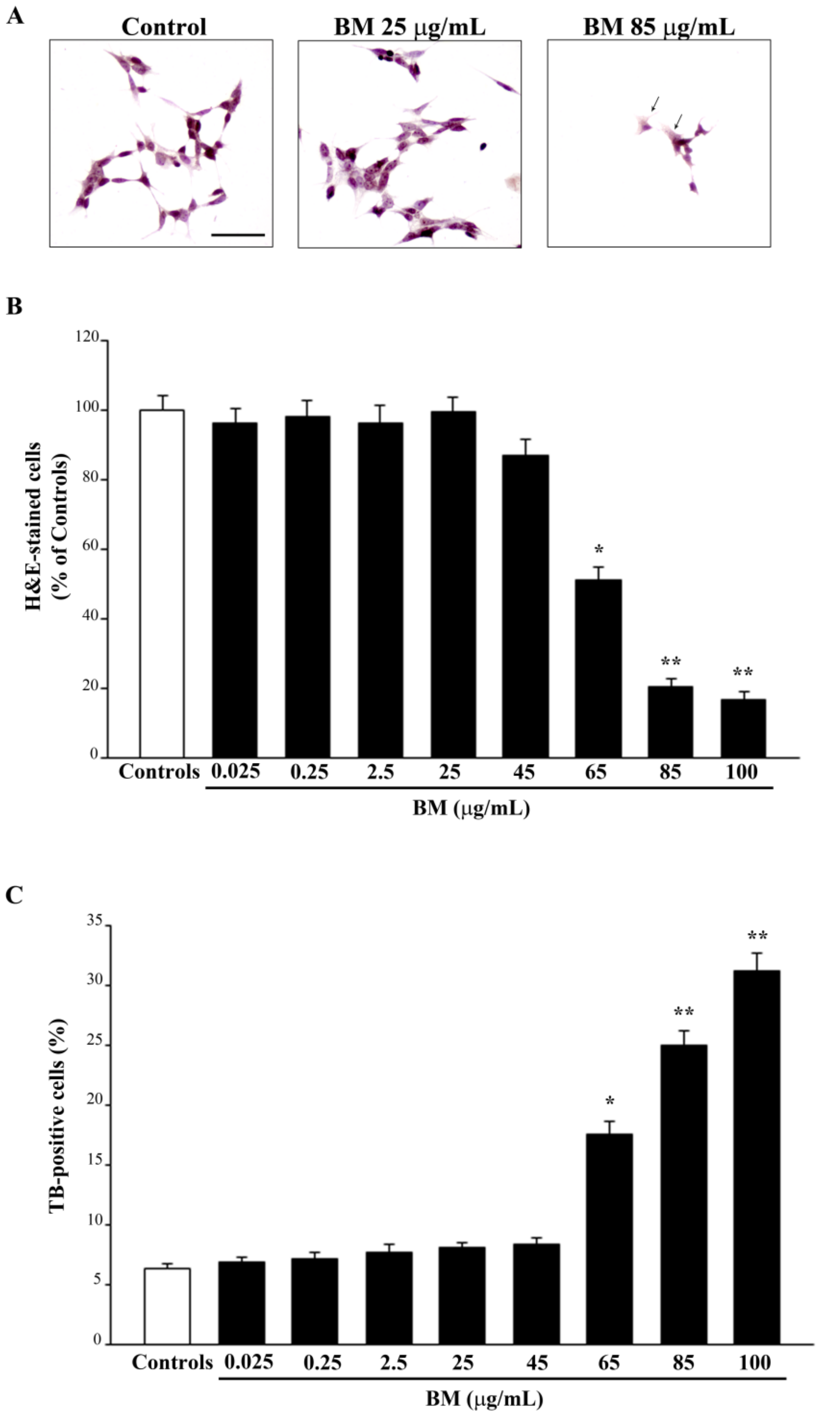

Supplementary Figure S1. Dose–response of BM on H&E- and TB-stained SH-SY5Ycells. (**A**) Representative H&E staining, following increasing doses of BM. Arrows indicate cell alterations induced by high doses of BM. Graphs report the count of (**B**) H&E- and (**C**) TB-stained cells following various doses of BM (from 0.025 μg/mL up to 100 μg/mL). Values are given as mean percentage±S.E.M. of cells from three independent experiments. *p< 0.05 compared with controls and BM up to 45 μg/mL; **p< 0.05 compared with controls and BM up to 65 μg/mL. Scale bar=38 μm.

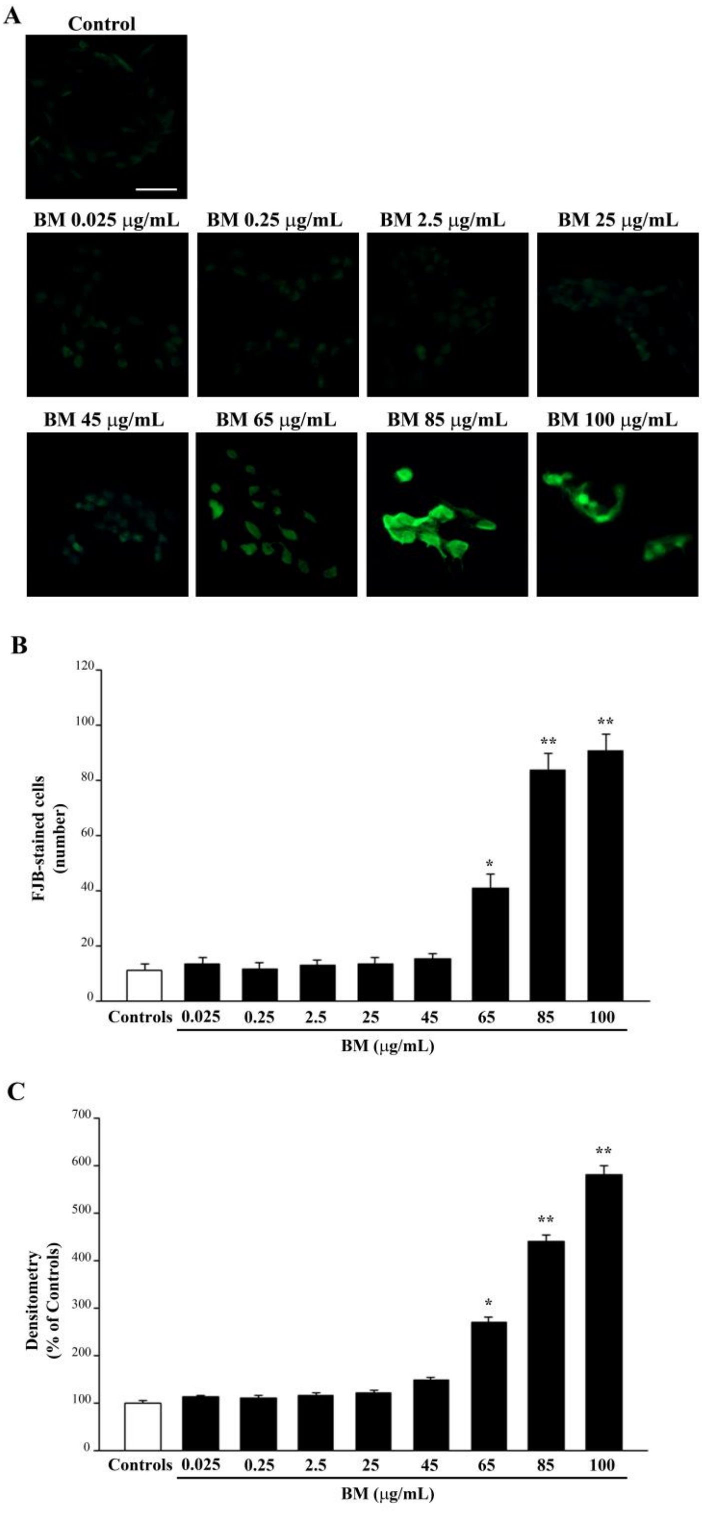

Supplementary Figure S2. Dose–response of BM on FJB staining in SH-SY5Ycells. (**A**) Representative pictures of FJB-stained PC12 cells after treatment with increasing doses of BM (from 0.025 μg/mL up to 100 μg/mL) for 72 h. Arrows indicate cells intensely stained with FJB. The number and the optical density of FJB fluorescent cells are reported in the graphs (**B**, **C**, respectively). Values are given as (**B**) mean±S.E.M. of FJB-positive cells and (**C**) mean percentage+S.E.M. of optical density (assuming Controls as 100% density) from three independent experiments. *$p < 0.05$ compared with controls and BM up to 45 μg/mL; **$p < 0.05$ com-pared with controls and BM up to 65 μg/mL. Scale bar=25 μm.

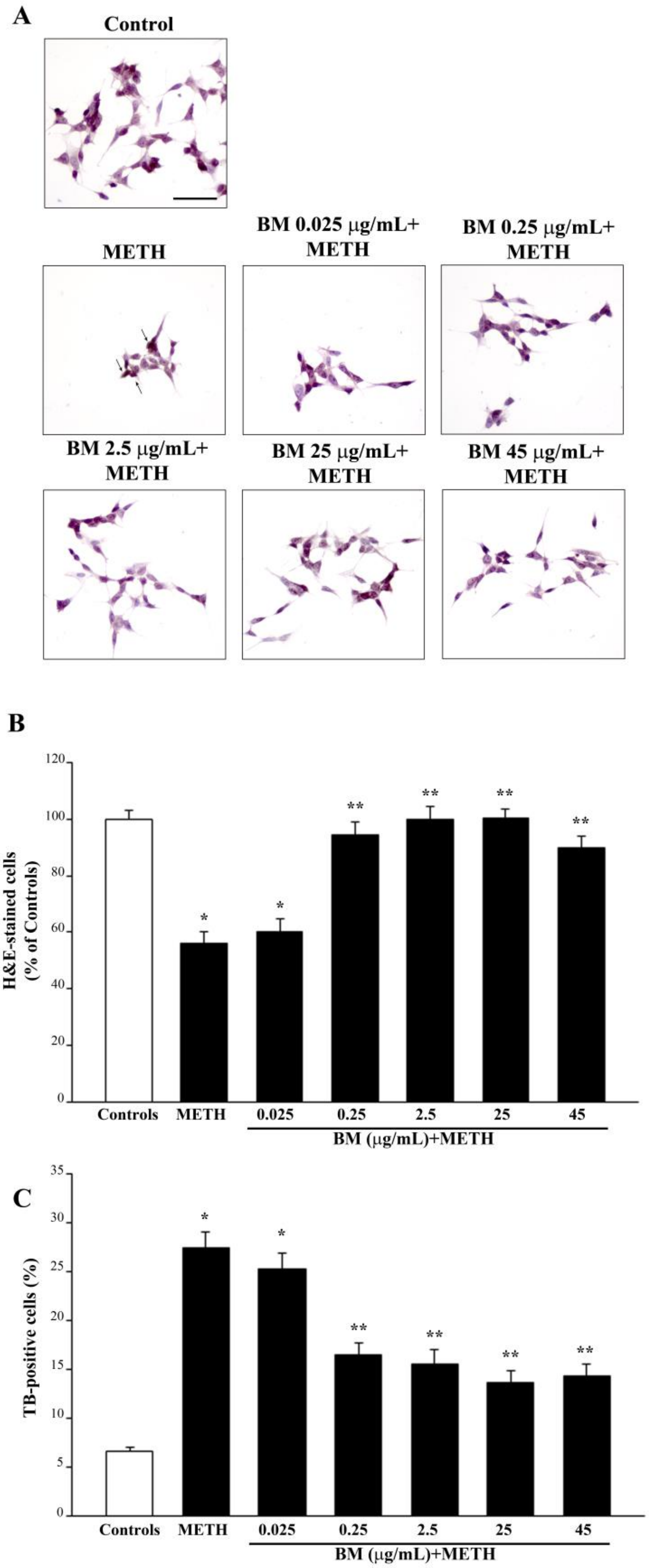

Supplementary Figure S3. BM reduces toxicity induced by METH in SH-SY5Ycells. (A) Representative H&E pictures of cells in baseline conditions (control) and following treatment with METH (100 μM), alone or in combination with various doses of BM (from 0.025 μg/mL up to 45 μg/mL). Arrows indicate cell alterations induced by METH. Graphs report the count of (**B**) H&E- and (**C**) TB-stained cells. Values are given as the mean percentage±S.E.M. of cells from three independent experiments. *p<0.05 compared with controls; **p< 0.05 compared with METH. Scale bar=38 μm.

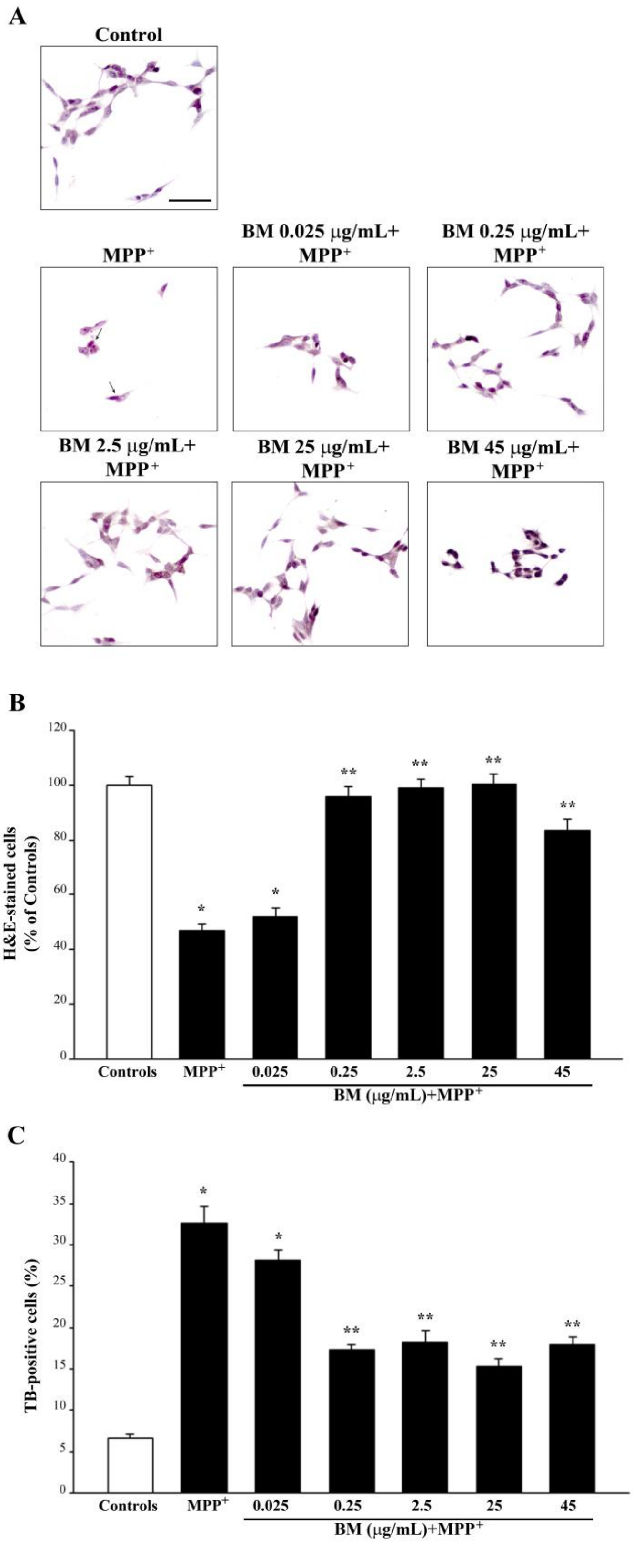

Supplementary Figure S4. BM reduces toxicity induced by MPP^+ in SH-SY5Ycells. (**A**) Representative H&E pictures of cells in baseline conditions (control) and following treatment with MPP^+ (100 μM), alone or in combination with various doses of BM (from 0.025 μg/mL up to 45 μg/mL). Arrows indicate cell alterations induced by MPP^+. Graphs report the count of (**B**) H&E- and (**C**) TB-stained cells. Values are given as the mean percentage±S.E.M. of cells counted in three independent experiments. *$p<0.05$ compared with controls; **$p<0.05$ compared with MPP^+. Scale bar=38 μm.

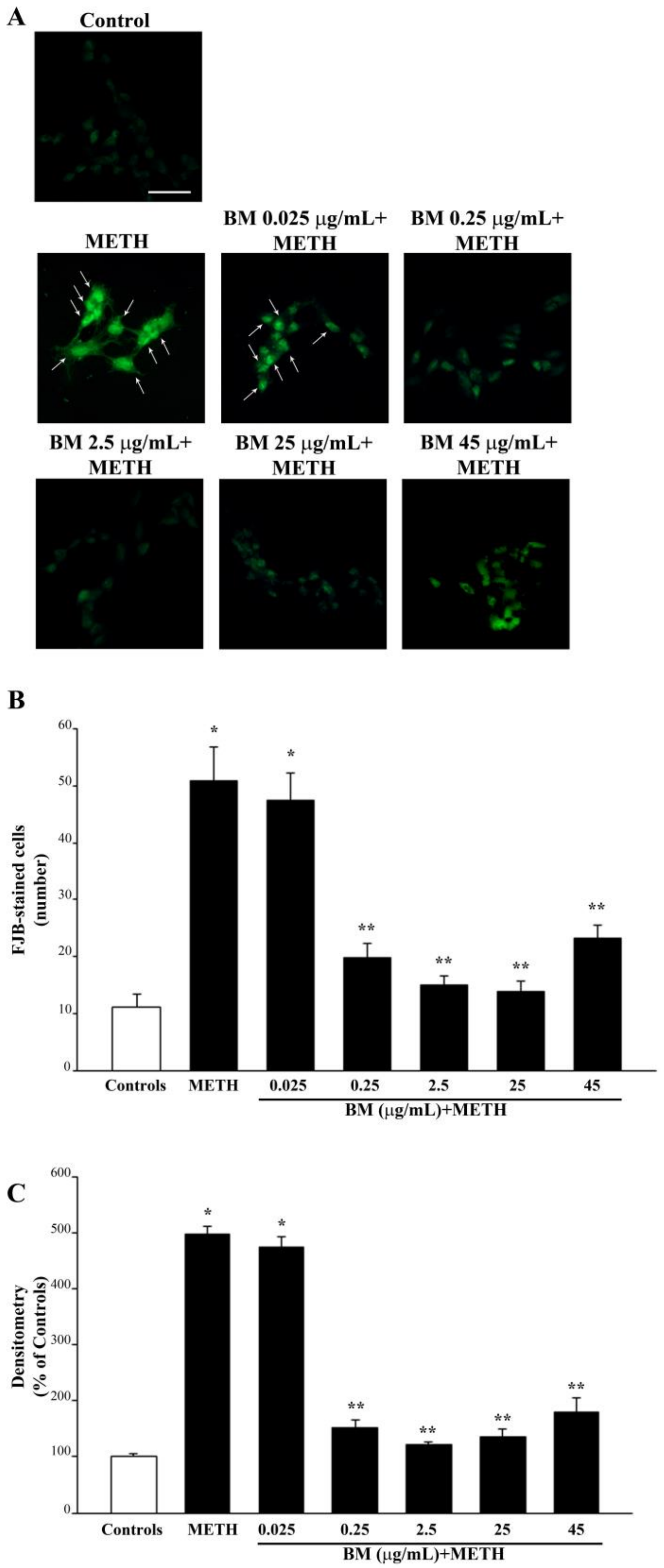

Supplementary Figure S5. BM decreases METH-induced FJB staining in SH-SY5Ycells. (**A**) Representative pictures of FJB-stained cells after METH (100 μM), alone or in combination with various doses of BM (from 0.025 μg/mL up to 45 μg/mL). Arrows indicate cells intensely stained with FJB. Graphs report the number (**B**) and the intensity (**C**) of FJB fluorescent cells. Values are given as mean ± S.E.M (**B**) or the mean percentage ± S.E.M. (assuming controls as 100%, **C**) of cells from three independent experiments. *p<0.05 compared with controls; **p<0.05 compared with METH. Scale bar=25 μm.

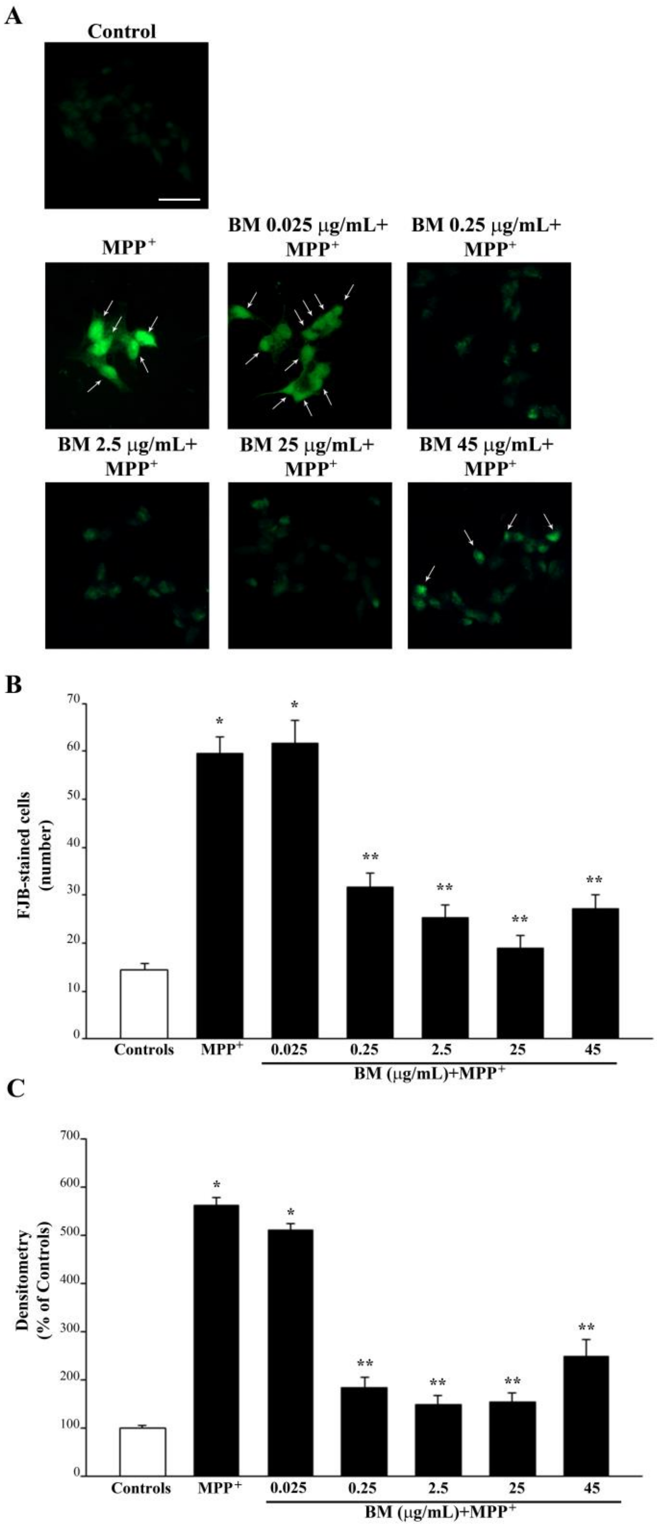

Supplementary Figure S6. BM decreases MPP+-induced FJB staining in SH-SY5Ycells. (**A**) Representative pictures of FJB-stained cells after MPP+ (100 μM), alone or in combination with various doses of BM (from 0.025 μg/mL up to 45 μg/mL). Arrows indicate cells intensely stained with FJB. Graphs report the number (**B**) and the intensity (**C**) of FJB fluorescent cells. Values are given as mean ± S.E.M (**B**) or the mean percentage ± S.E.M. (assuming controls as 100%, **C**) of cells from three independent experiments. *p<0.05 compared with controls; **p<0.05 compared with METH. Scale bar=25 μm.

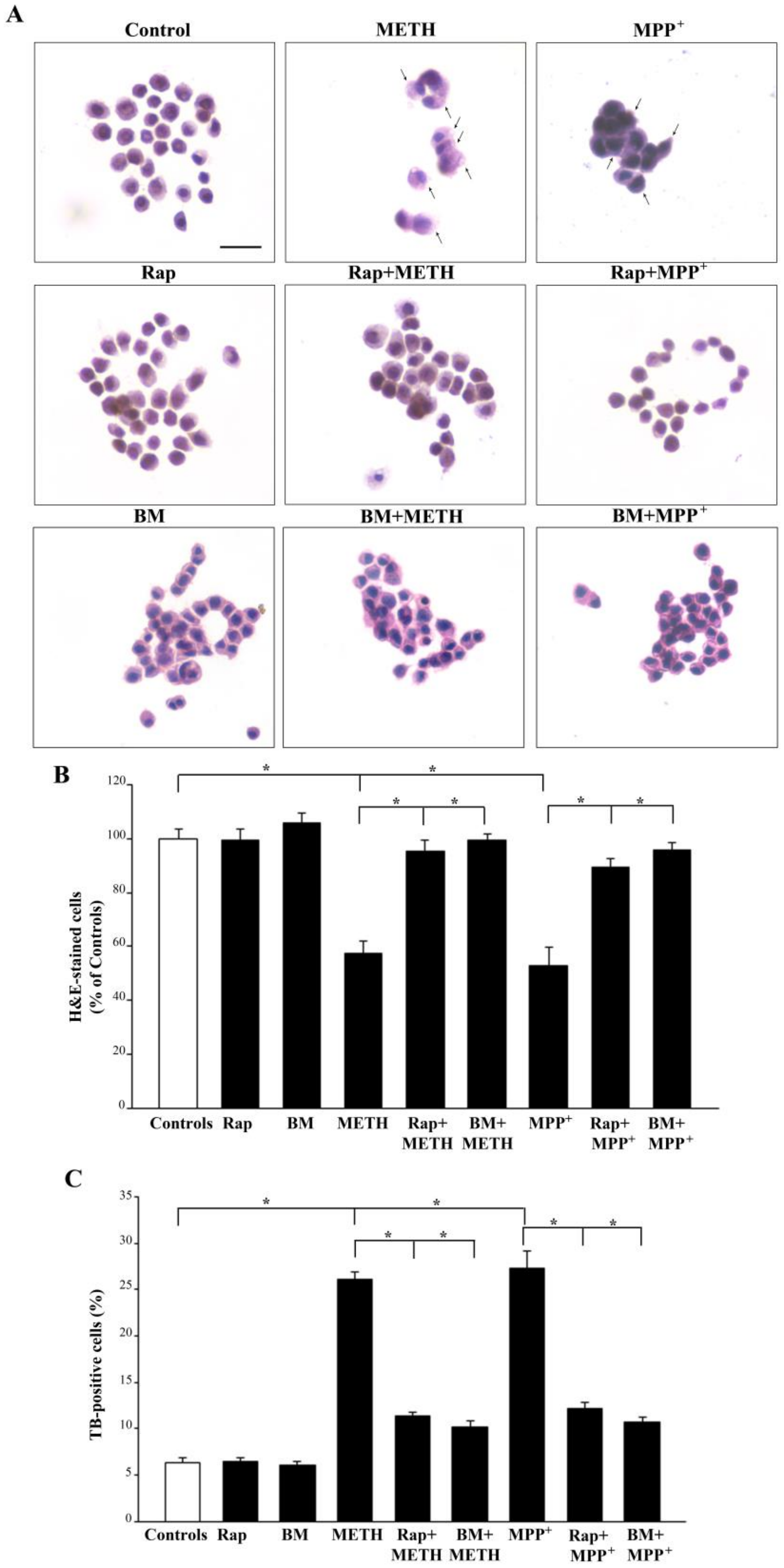

Supplementary Figure S7. BM and rapamycin produce comparable neuroprotective effects against METH- and MPP+-induced toxicity in PC12 cells. (**A**) Representative pictures of H&E-stained cells in control conditions and following treatment with METH (100 μM) or MPP+ (100 μM), alone or combined with rapamycin (Rap, 100 nM) or BM (25 μg/mL). Arrows indicate METH- or MPP+-induced morphological alterations. Graphs report the count of (**B**) H&E- and (**C**) TB-stained cells. Values are given as the mean percentage±S.E.M. of cells from three independent experiments. *p<0.05. Scale bar= 20 μm.

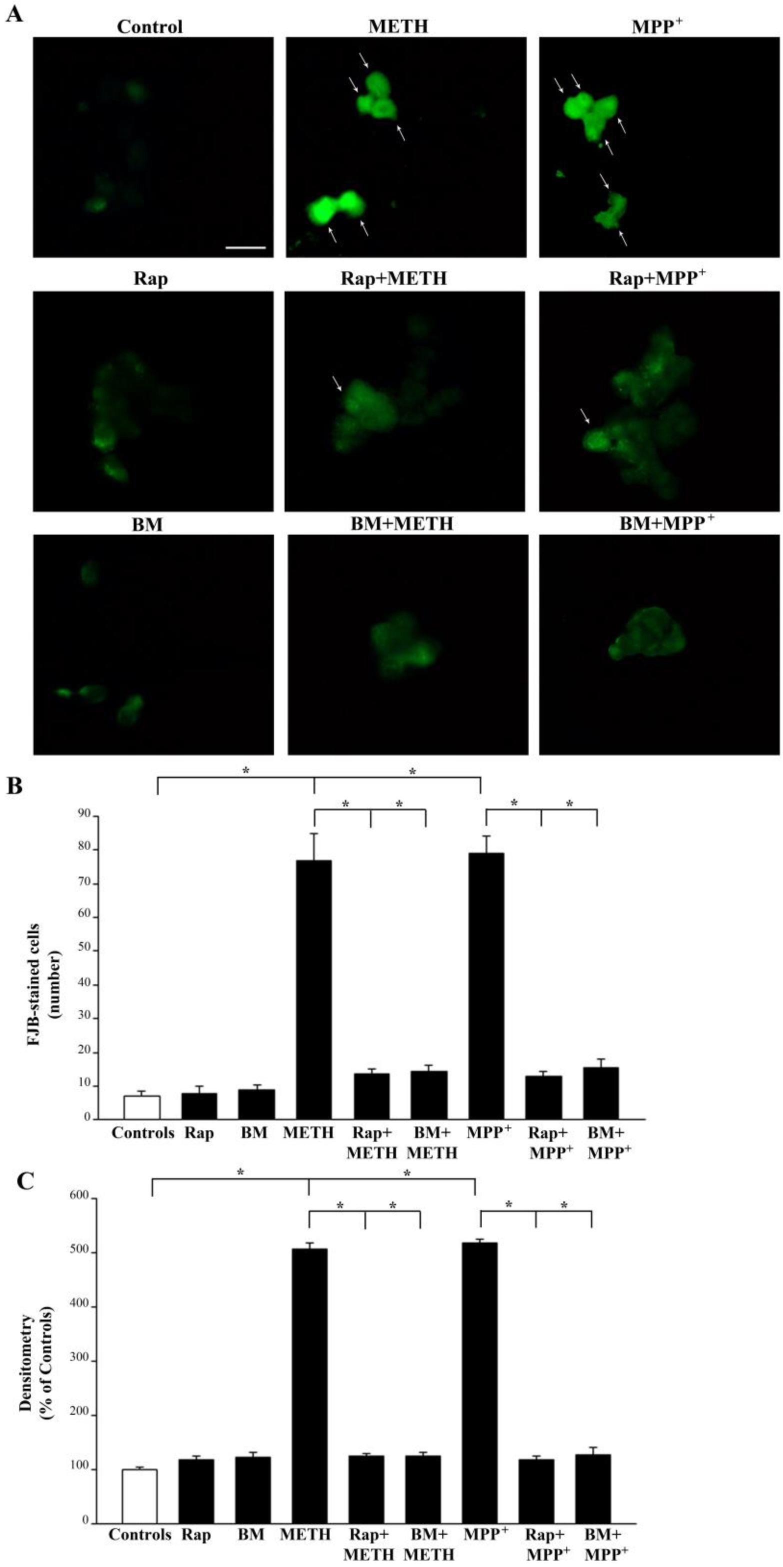

Supplementary Figure S8. BM and rapamycin produce comparable reduction of FJB histo-fluorescence induced by METH- or MPP$^+$ in PC12 cells. (**A**) Representative pictures of FJB-stained cells in control conditions and following METH (100 μM) or MPP$^+$ (100 μM) treatment, alone or combined with rapamycin (Rap, 100 nM) or BM (25 μg/mL). Arrows indicate cells intensely stained with FJB. Graphs report the number (**B**) and the intensity (**C**) of FJB fluorescent cells. Values are given as mean ± S.E.M (**B**) or the mean percentage ± S.E.M. (assuming controls as 100%, **C**) of cells from three independent experiments. *p<0.05. Scale bar=20 μm.

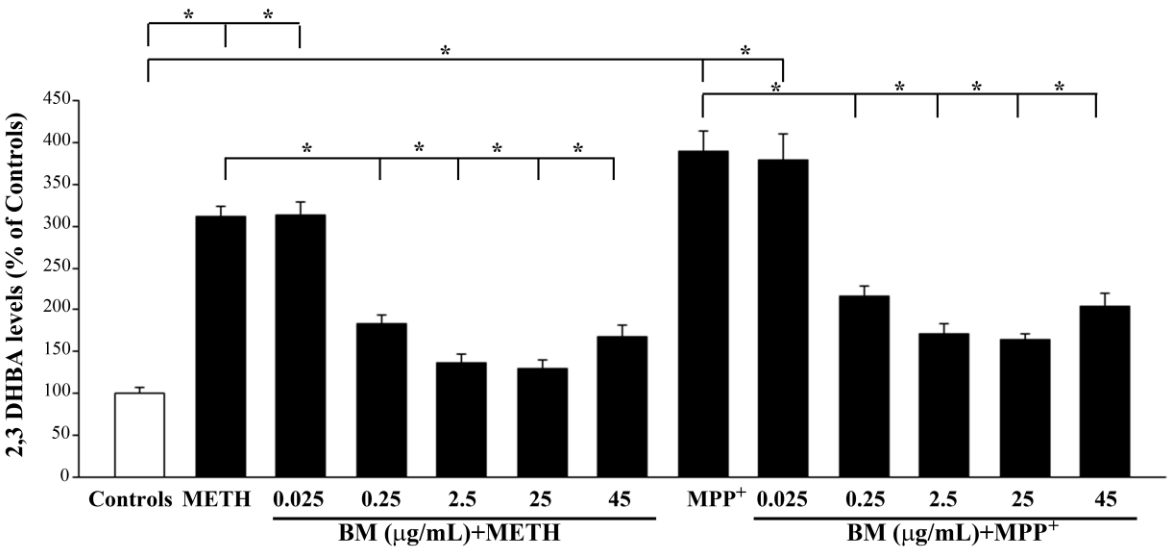

Supplementary Figure S9. Low doses of BM decrease METH- and MPP^+-induced ROS formation in SH-SY5Y cells. The graph reports the levels of 2,3 DHBA produced by combined treatment of BM and METH or MPP^+. Values are given as the mean percentage ± S.E.M. of controls (assuming controls as 100% density). Data are obtained from three independent experiments. *p<0.05.

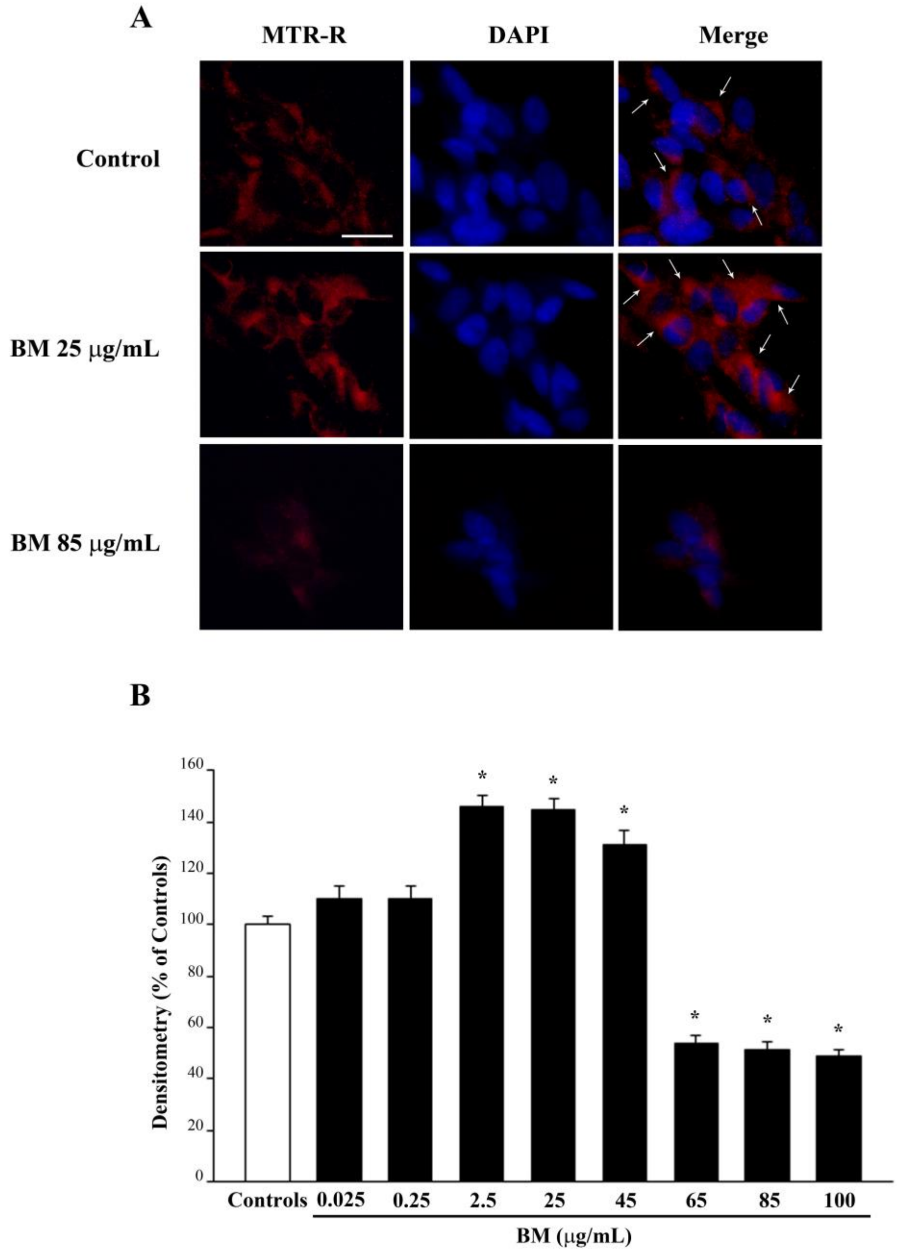

Supplementary Figure S10. MitoTracker Red (MTR-R) fluorescence after increasing doses of BM in SH-SY5Y cells. (**A**) Representative pictures show that low doses of BM, at the protective dose of 25 μg/mL, produces an increase of MTR-R fluorescence, while a frankly toxic dose of BM (85 μg/mL) decreases MTR-R fluorescence below of control. Arrows indicate intensely MTR-R-stained cells. Cell nuclei are stained in blue with DAPI. (**B**) The graph reports the densitometry of MTR-R fluorescence measured in the full range of BM doses used in this work (from0.025 μg/mL up to 100 μg/mL). Values are given as mean percentage±S.E.M. of optical density (assuming controls as 100%) from three independent experiments. *p<0.05 compared with controls. Scale bar=13 μm.

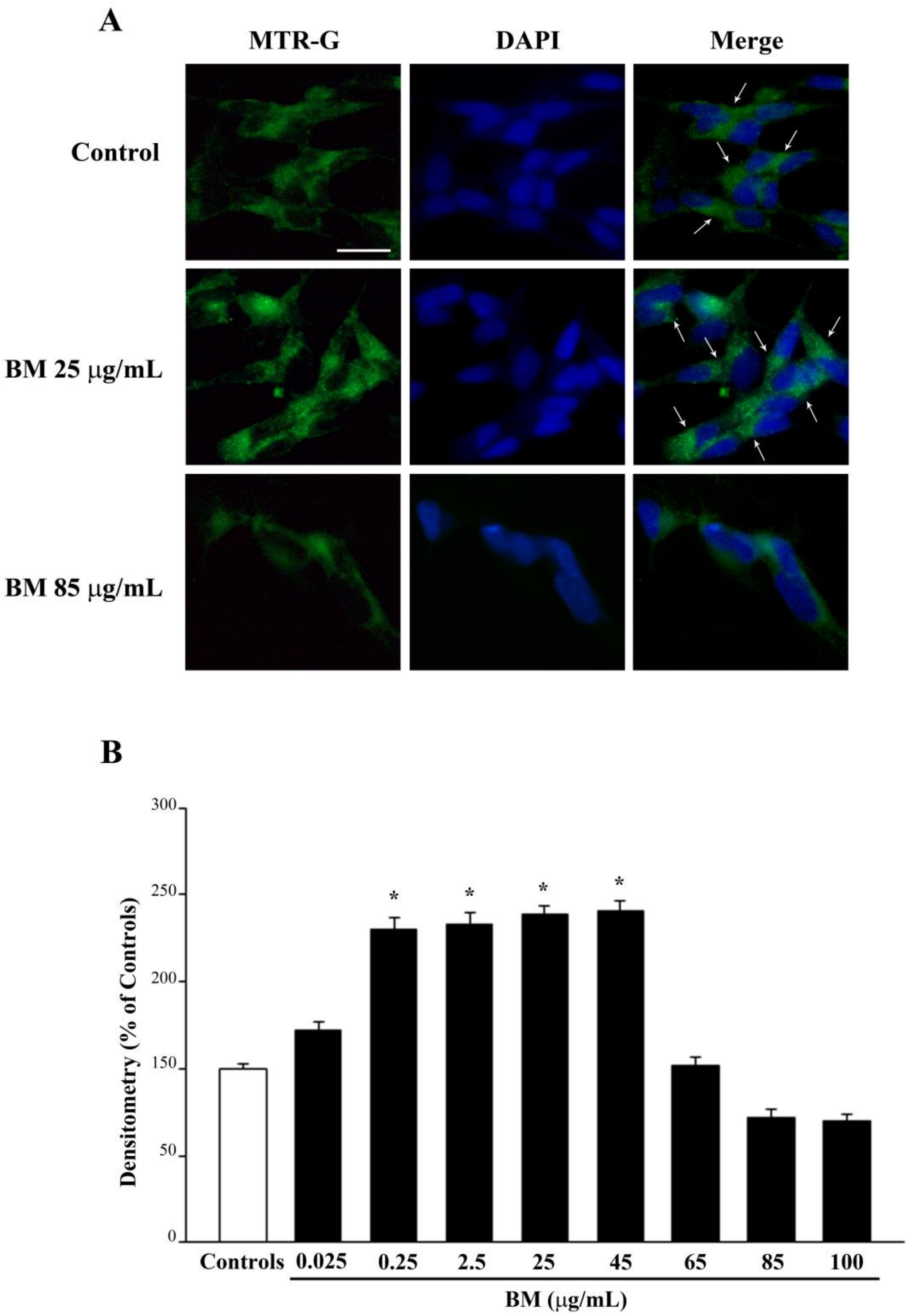

Supplementary Figure S11. MitoTracker Green (MTR-G) fluorescence after increasing doses of BM in SH-SY5Y cells. (**A**) Representative pictures show that MTR-G fluorescence is increased by the protective dose of BM (25 μg/mL), while it is reduced by a frankly toxic dose of BM (85 μg/mL). Arrows indicate intensely MTR-G-stained cells. Cell nuclei are stained in blue with DAPI. (**B**) The graph reports the densitometry of MTR-G fluorescence measured in the full range of BM doses used in this work (from0.025 μg/mL up to 100 μg/mL). Values are given as mean percentage±S.E.M. of optical density

(assuming controls as 100%) obtained from three independent experiments. *$p<0.05$ compared with controls. Scale bar=13 μm.

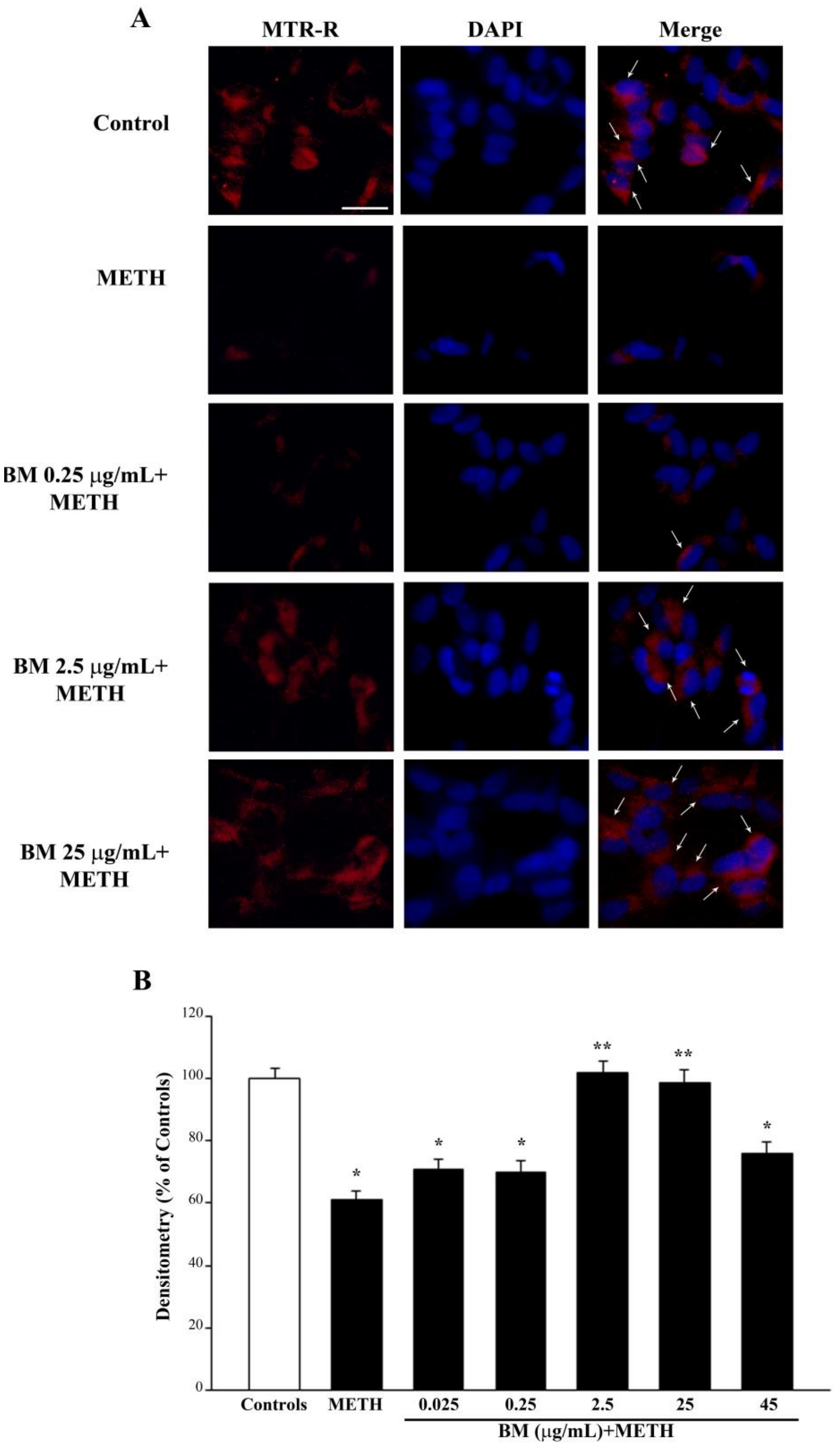

Supplementary Figure S12. MitoTracker Red (MTR-R) fluorescence after combined treatments with BM and METH in SH-SY5Y cells. (**A**) Representative pictures of MTR-R fluorescence show that BM counteracts the reduction of MTR-R

fluorescence induced by METH. Arrows indicate intensely MTR-R fluorescence. Cell nuclei are stained in blue with DAPI. (**B**) The graph reports the MTR-R fluorescence intensity measured for the neuroprotective doses of BM (from 0.025 μg/mL to 45 μg/mL). Note that the protective effect of BM against METH-induced mitochondrial toxicity, is evident at the doses of 2.5 μg/mL and 25 μg/mL. Values are given as mean percentage±S.E.M. of optical density (assuming controls as 100%) from each experimental group from three independent experiments. *$p<0.05$ compared with controls; **$p<0.05$ compared with METH. Scale bar=13 μm.

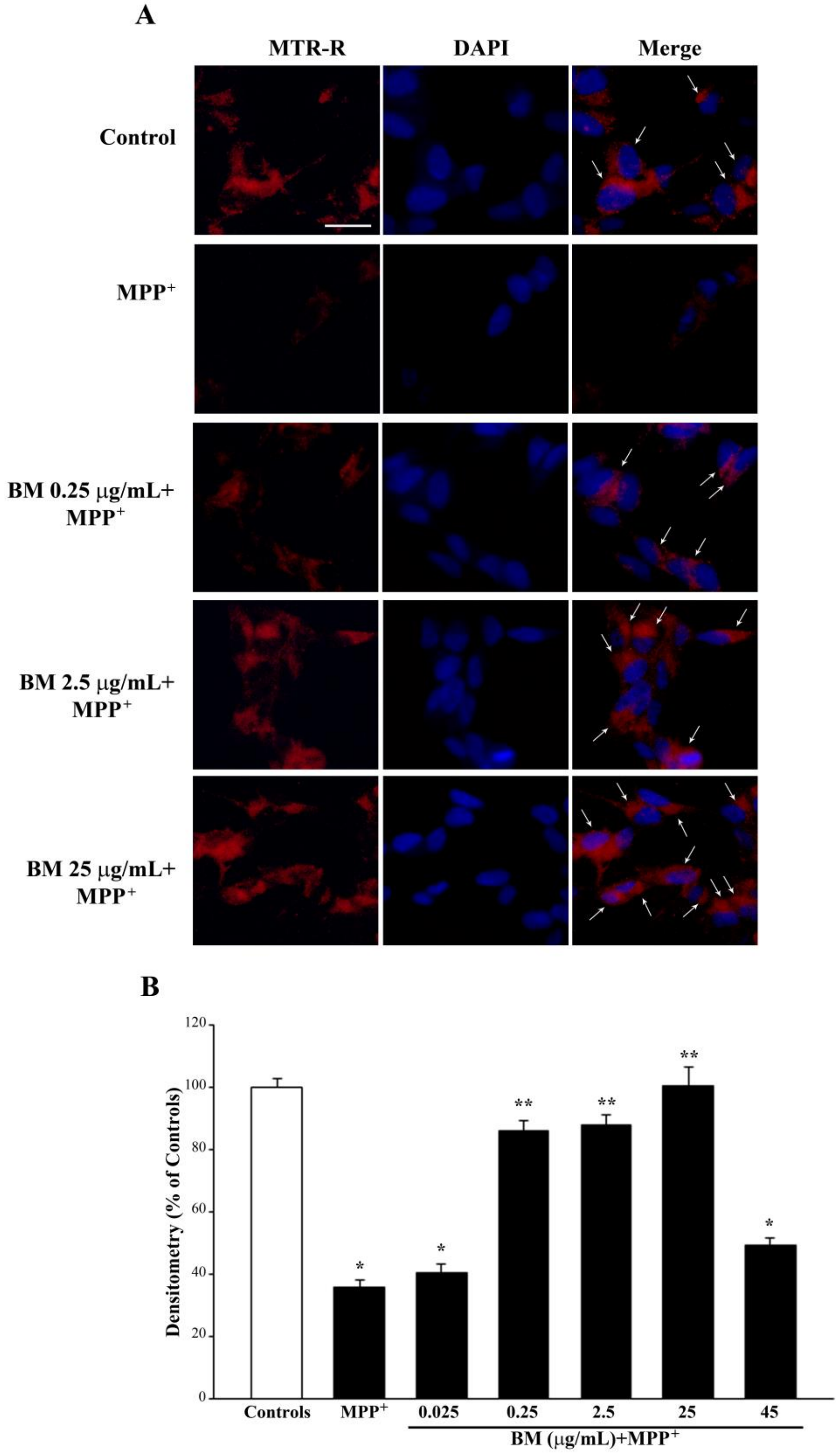

Supplementary Figure S13. MitoTracker Red (MTR-R) fluorescent SH-SY5Y cells after combined administration of BM and MPP+. (**A**) Representative pictures of MTR-R fluorescence show that BM is protective against the reduction of MTR-R staining induced by MPP+. Arrows indicate intensely MTR-R fluorescence. Cell nuclei are stained in blue with DAPI. (**B**) The graph reports the MTR-R fluorescence intensity. Note that the protective effect of BM against the MPP+-induced mitochondrial toxicity is already evident for the dose of 0.25 μg/mL. Values are given as the mean percentage±S.E.M. of optical density (assuming controls as 100%) from each experimental group obtained from three independent experiments. *p<0.05 compared with controls; **p<0.05 compared with MPP+. Scale bar=13 μm.

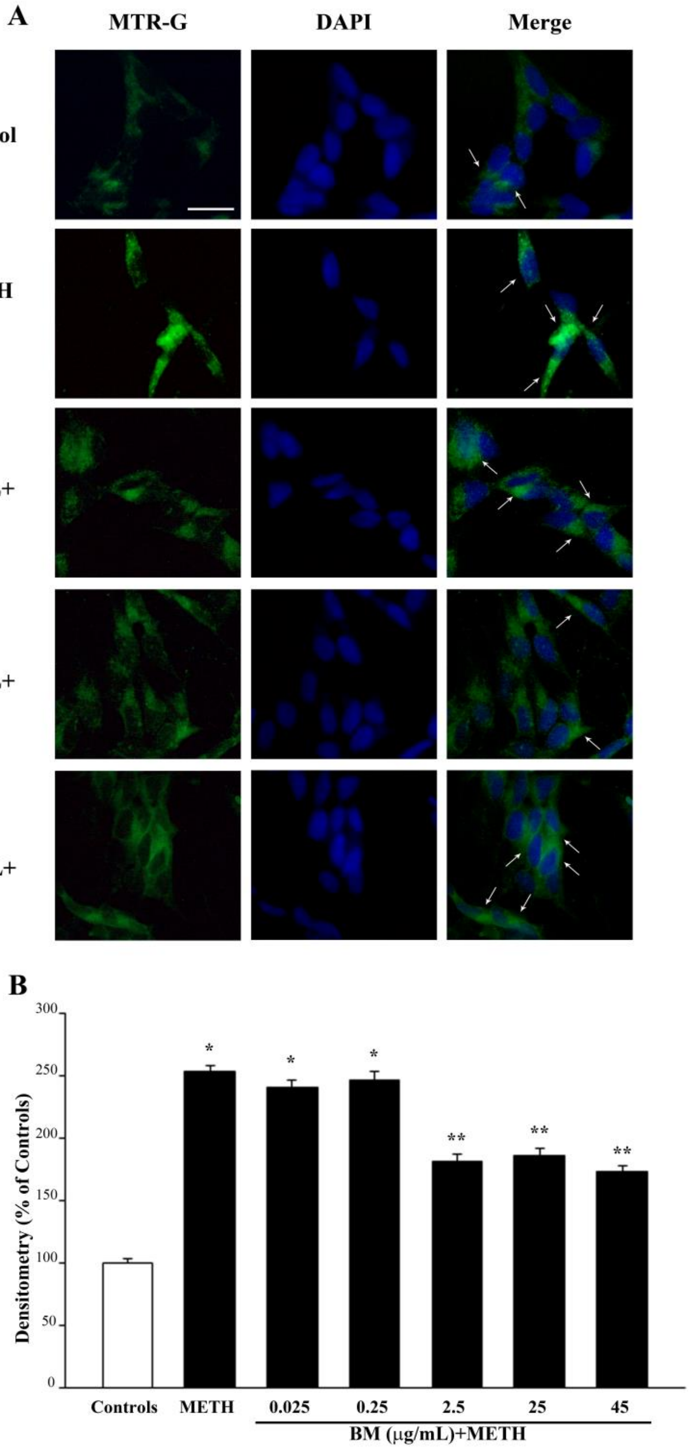

Supplementary Figure S14. MitoTracker Green (MTR-G) fluorescent SH-SY5Y cells after combined administration of BM and METH. (**A**) Representative pictures show that METH increases MTR-G fluorescence compared with control. This effect is less pronounced when METH is given in combination with BM. Cell nuclei are stained in blue with DAPI. The graph in

(**B**), which reports MTR-G fluorescence intensity, indicates that this effect of BM on METH-induced increase of MTR-G fluorescence is evident from the dose of 2.5 μg/mL up to the dose of 45 μg/mL. Values are given as mean percentage±S.E.M. of optical density (assuming controls as 100%) from each experimental group from three independent experiments. *$p<0.05$ compared with controls; **$p<0.05$ compared with METH. Scale bar=13 μm.

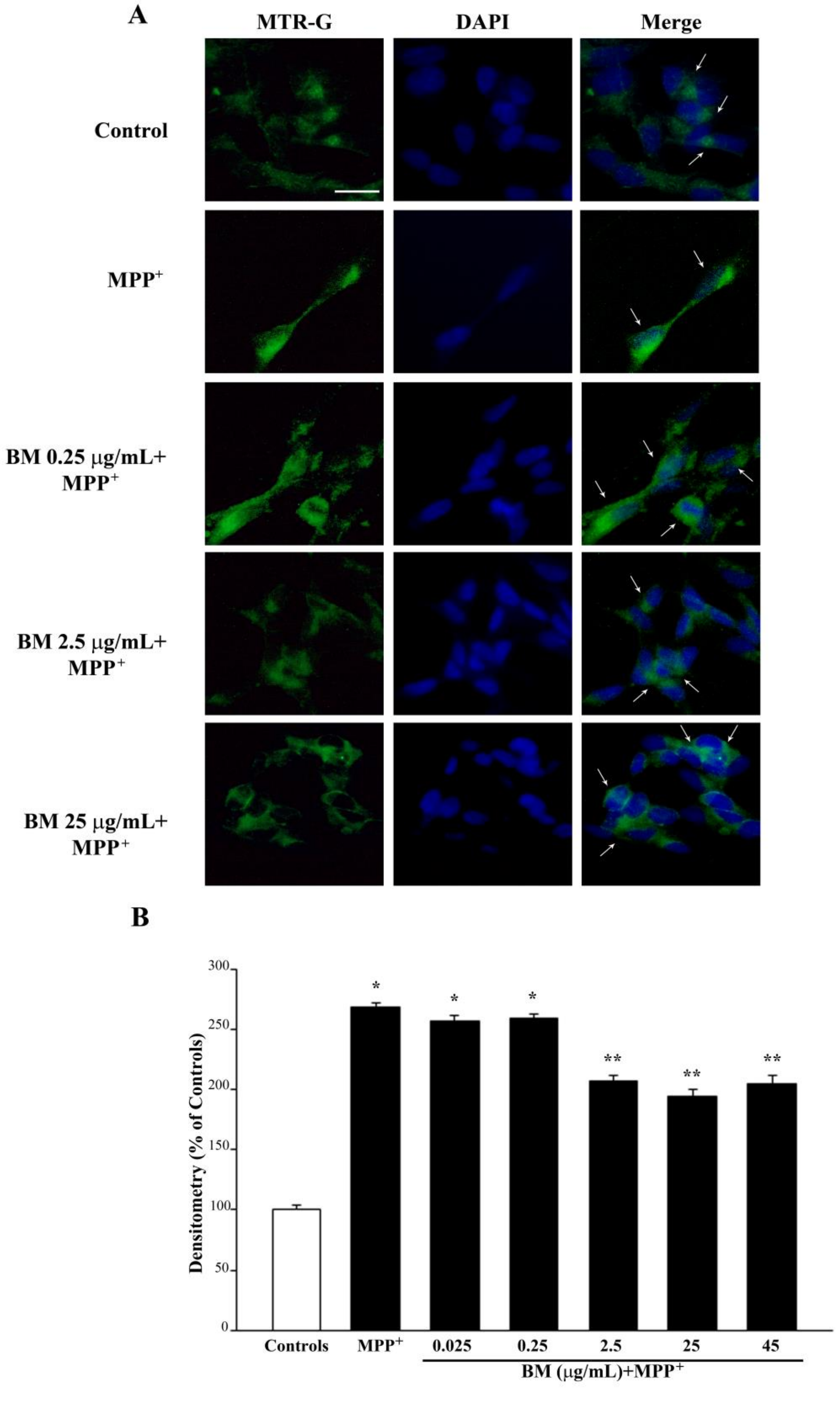

Supplementary Figure S15. MitoTracker Green (MTR-G) fluorescent SH-SY5Y cells after combined administration of BM and MPP+. (**A**) Representative pictures of MTR-G fluorescence show that MPP+ increases MTR-G fluorescence compared with control. This effect is less pronounced when MPP+ is given in combination with BM. Cell nuclei are stained in blue with DAPI. The graph in (**B**), which reports the measures of the MTR-G fluorescence intensity, indicates that this effect of BM on MPP+-induced increase of MTR-G fluorescence is evident from the dose of 2.5 µg/mL up to the dose of 45 µg/mL. Values are given as mean percentage±S.E.M. of optical density (assuming controls as 100%) from each experimental group from three independent experiments. *$p<0.05$ compared with controls; **$p<0.05$ compared with MPP+. Scale bar=13 µm.

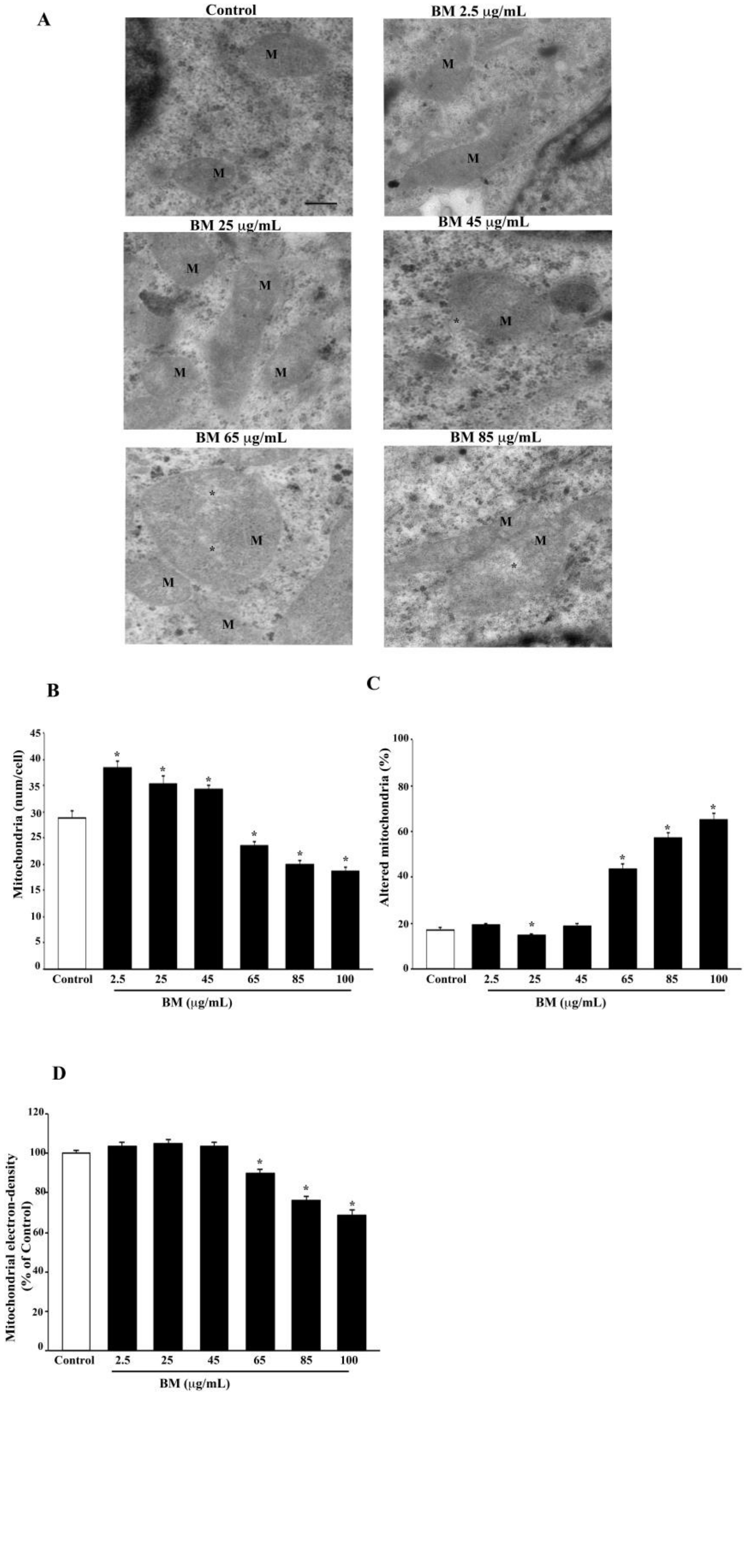

Supplementary Figure S16. Effects of increasing doses of BM on mitochondrial ultrastructure of SH-SY5Y cells. (**A**) Representative micro-graphs show that BM at low doses (i.e. 2.5 µg/mL and 25 µg/mL) preserves the ultrastructure of mitochondria. In contrast, at the highest doses used (ranging from 65 µg/mL to 85 µg/mL) BM induces mitochondrial alterations consisting in matrix dilution (*) and fragmented cristae. M=mitochondria. The graphs report the effects of increasing doses of BM on: (**B**) the number of total mitochondria per cell, (**C**) the percentage of altered mitochondria per cell, and (**D**) the electron-density of mitochondrial matrix. Values are given as the mean±S.E.M. (**B**), mean percentage±S.E.M. (**C** and **D**) of counts from three independent experiments. *p<0.05 compared with controls. Scale bar=280 nm.

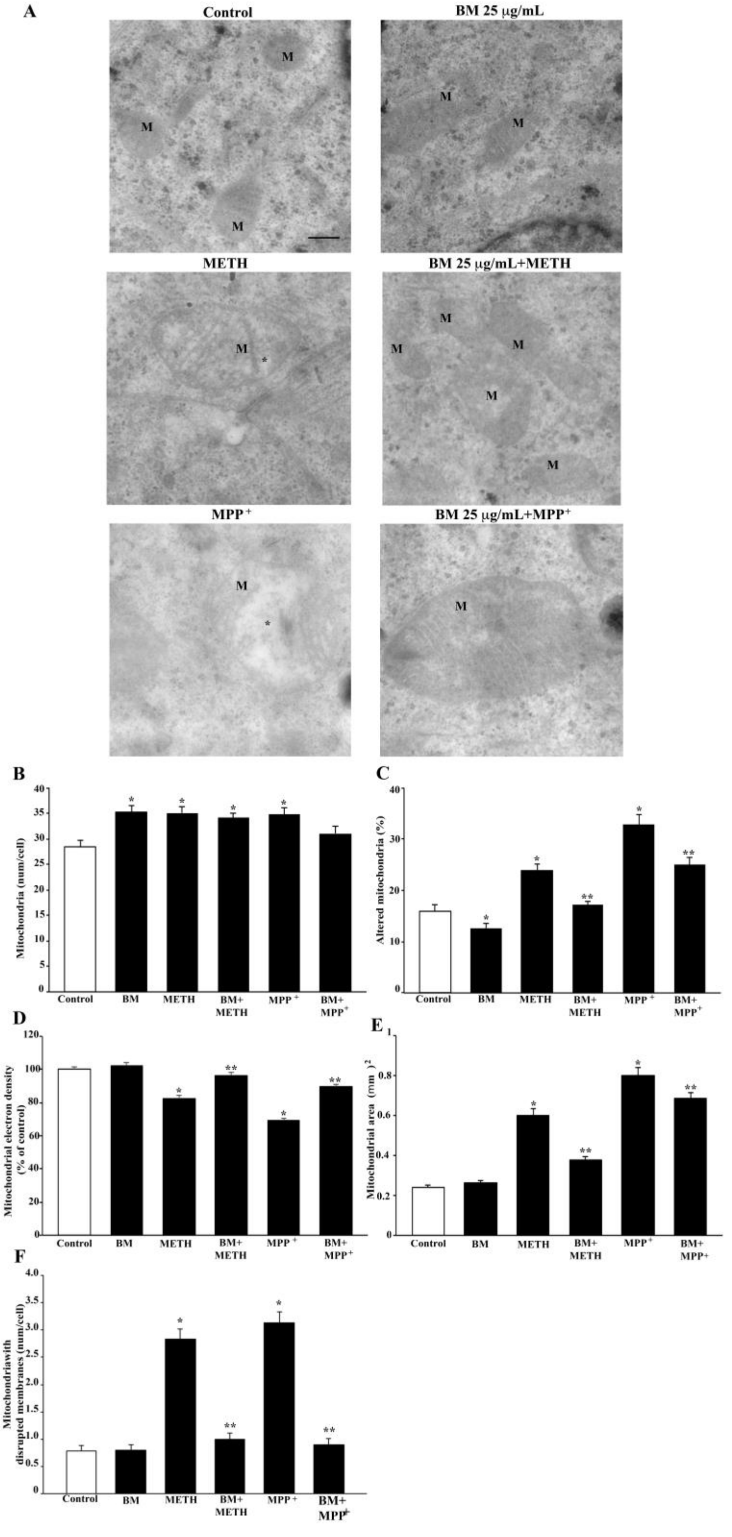

Supplementary Figure S17. Effects of combined treatment with BM (25 μg/mL) and METH or MPP+ on mitochondrial ultrastructural morphometry of SH-SY5Y cells. (**A**) Representative micrographs of mitochondria show that METH induces mitochondrial alteration, consisting of matrix dilution (*) and fragmented cristae, which are even more severe after MPP+, thus confirming what observed in PC12 cells. Pre-treatment with BM (25 μg/mL) counteracts the mitochondrial alterations induced by both neurotoxins. Graphs report the effects of combined BM (25 μg/mL) and METH or MPP+ treatment on the number of total mitochondria per cell (**B**), the percentage of altered mitochondria per cell (**C**), the electron-density of the mitochondrial matrix (**D**) the mitochondrial area (**E**) and the number of mitochondria with disrupted membranes (**F**). Values are given as the mean±S.E.M. (**B**, **E** and **F**), or mean percentage±S.E.M. (**C** and **D**) of measures from three independent experiments. *$p<0.05$ compared with controls; **$p<0.05$ compared with METH or MPP+. M=mitochondria. Scale bar=280 nm.

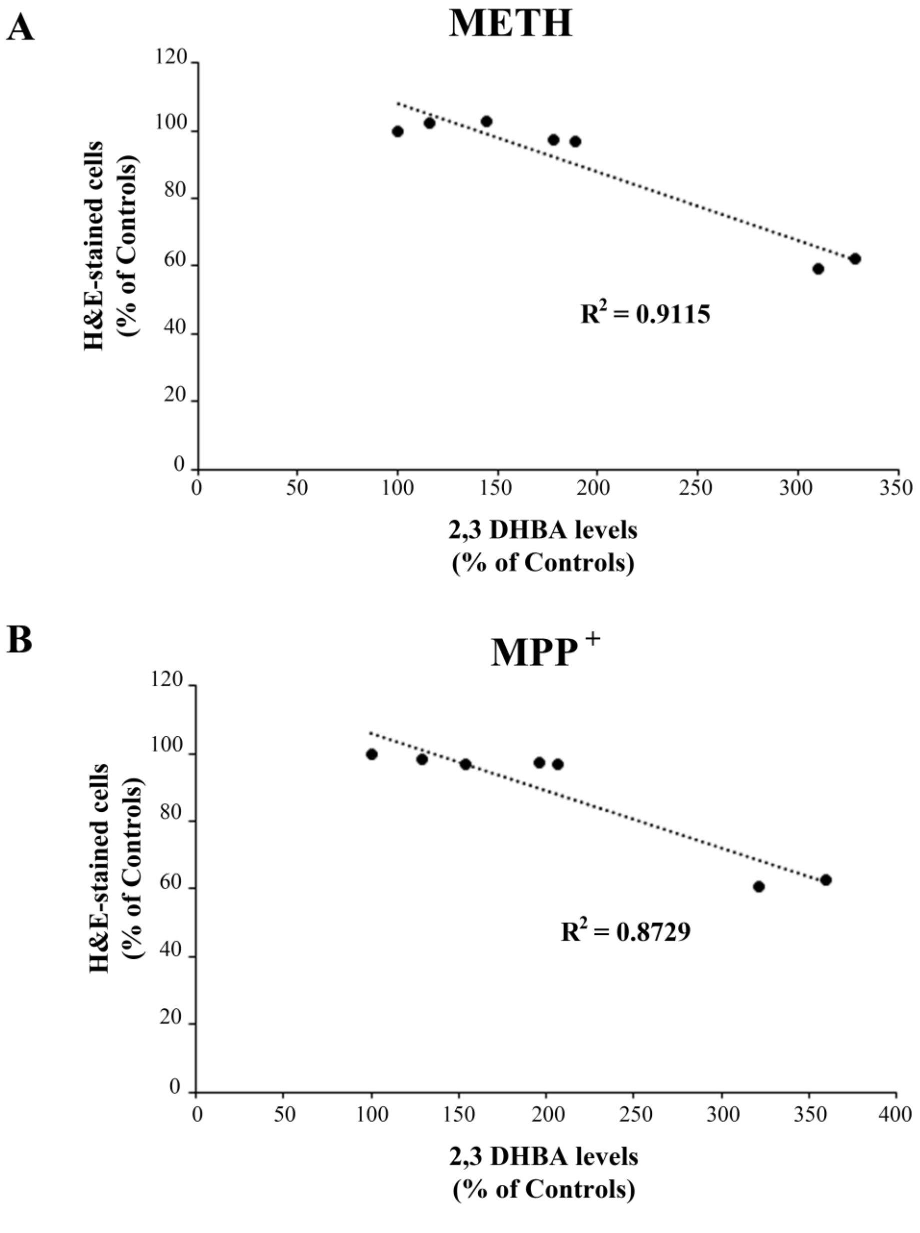

Supplementary Figure S18. Direct correlation between 2,3 DHBA levels and METH- or MPP^+-induced toxicity when using BM. The graphs report the linear regression between the percentage of H&E-positive cells and the percentage of 2,3 DHBA levels, which are measured following METH (100 μM, **A**) or MPP^+ (100 μM, **B**) exposure for 72 h . This correlation is higher after METH treatments (p=0.0008) compared with MPP^+ treatments (p=0.0021).

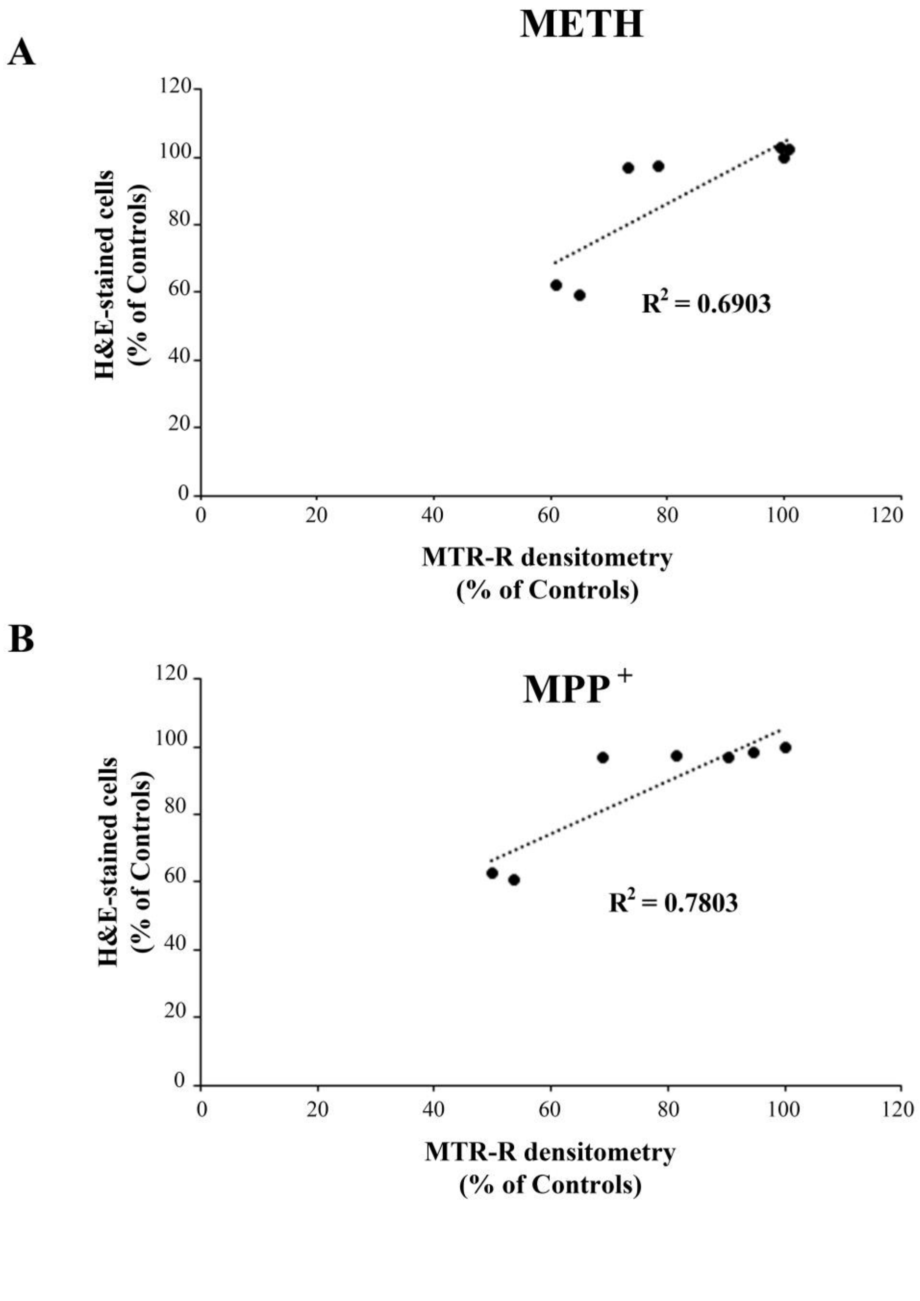

Supplementary Figure S19. Direct correlation between MTR-R densitometry and METH- or MPP^+-induced toxicity when using BM. The graphs report the linear regression between the percentage of H&E-positive cells and the percentage of

MTR-R densitometry, which are measured following METH (100 μM, **A**) or MPP^+ (100 μM, **B**) exposure for 72 h . This correlation is higher after MPP^+ treatments (*p*=0.0084) compared with METH treatments (*p*=0.0206).
